# YAP nuclear translocation induced by HIF-1α prevents DNA damage under hypoxic conditions

**DOI:** 10.1038/s41420-023-01687-5

**Published:** 2023-10-20

**Authors:** Heng-Ai Chang, Rui-Zhi Ou Yang, Jing-Ming Su, Thi My Hang Nguyen, Junne-Ming Sung, Ming-Jer Tang, Wen-Tai Chiu

**Affiliations:** 1 Institute of Basic Medical Sciences, National Cheng Kung University, Tainan, 701 Taiwan, ROC; 2 Department of Biomedical Engineering, National Cheng Kung University, Tainan, 701 Taiwan, ROC; 3 Department of Internal Medicine, National Cheng Kung University Hospital, Tainan, 701 Taiwan, ROC; 4 Institute of Clinical Medicine, College of Medicine, National Cheng Kung University, Tainan, 701 Taiwan, ROC; 5 Department of Physiology, College of Medicine, National Cheng Kung University, Tainan, 701 Taiwan, ROC; 6 International Center for Wound Repair and Regeneration, National Cheng Kung University, Tainan, 701 Taiwan, ROC; 7 Medical Device Innovation Center, National Cheng Kung University, Tainan, 701 Taiwan, ROC

**Keywords:** Macroautophagy, Nephrons, DNA, HIPPO signalling, Extracellular signalling molecules

## Abstract

Maladaptive repair of acute kidney injury (AKI) is associated with a high risk of developing chronic kidney disease deemed irremediable even in present days. When AKI arises from ischemia-reperfusion injury, hypoxia usually plays a major role. Although both hypoxia-inducible factor-1α (HIF-1α) and yes-associated protein (YAP) have been proven to promote renal cell survival under hypoxia, there is a lack of research that studies the crosstalk of the two and its effect on kidney repair. In studying the crosstalk, CoCl_2_ was used to create a mimetic hypoxic environment. Immunoprecipitation and proximity ligation assays were performed to verify protein interactions. The results show that HIF-1α interacts with YAP and promotes nuclear translocation of YAP at a high cell density under hypoxic conditions, suggesting HIF-1α serves as a direct carrier that enables YAP nuclear translocation. This is the first study to identify HIF-1α as a crucial pathway for YAP nuclear translocation under hypoxic conditions. Once translocated into a nucleus, YAP protects cells from DNA damage and apoptosis under hypoxic conditions. Since it is unlikely for YAP to translocate into a nucleus without HIF-1α, any treatment that fosters the crosstalk between the two holds the potential to improve cell recovery from hypoxic insults.

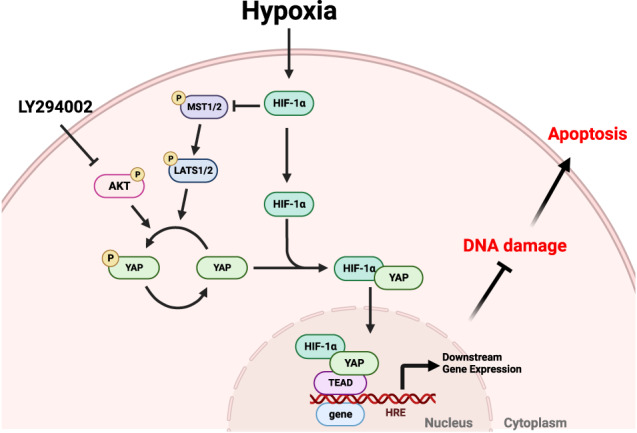

## Introduction

Ischemia-reperfusion injury (IRI) is a major cause of acute kidney injury (AKI). When kidneys suffer ischemia, both the oxygen flow and nutrient distribution to the kidneys would be severely restricted, resulting in the major symptoms of AKI, namely a sudden loss of kidney function and kidney damage. AKI is the single most important risk factor for chronic kidney disease (CKD). Even patients who recover from AKI have a higher risk of developing CKD later in life [1]. CKD is characterized by a progressive increase in renal fibrosis and loss of kidney function [2–4]. Currently, CKD remains irremediable and can only be prevented early on at the beginning stage of AKI. Patients who fail to recover from AKI will have to depend on dialysis to eliminate metabolic waste and maintain body fluid homeostasis throughout their lives. However, dialysis not only is expensive, but can also trigger various complications. Therefore, an effective method for the treatment of AKI is urgently required.

Often considered an essential prerequisite for AKI, hypoxia has also been found to accompany renal fibrosis and CKD [5]. It promotes the expression of hypoxia-inducible factor 1α (HIF-1α), the first pathway activated to regulate hypoxic responses [6]. By contrast, enriched oxygen levels allow prolyl hydroxylase domain protein to consistently hydroxylate HIF-1α at specific proline residues [7]. The hydroxylated prolines of HIF-1α are then identified and ubiquitinated by the von Hippel-Lindau protein, a tumor suppressor and E3 ubiquitin ligase. After that, HIF-1α is transported to the proteasome for the final degradation. The degradation of HIF-1α can happen within minutes, accounting for its short half-life (5 min) under normoxic conditions. Conversely, not only does HIF-1α become stabilized under hypoxic conditions, it also translocates to the nucleus and forms a heterodimer with HIF-1β. Once the HIF-1 complex is form, it recruits CBP/p300 co-activators and then binds to the hypoxia response element to transactivate downstream target genes [8, 9]. With HIF-1α initiating the upregulation of these genes, they kick in to boost angiogenesis, glycolytic energy metabolism, as well as cell proliferation, and ultimately help cell survive hypoxia. In an AKI mice model, as the subjects underwent 4 cycles of 8-min pre-exposure to hypoxia with 5-min intervals in between, renal functions are found protected and kidney injury attenuated in the 40-min IR surgery that follows, suggesting HIF-1α plays a preventive role against hypoxia [10, 11]. Although there are studies that investigate how hypoxia activates pro-apoptotic transcription factor, p53, and consequently lead to tubular cell death in vitro, the results were never discussed against the backdrop of HIF-1α activity, which usually drops as the duration of hypoxia lengthens [12, 13]. The literature on hypoxia-induced AKI has provided substantial data for us to consider HIF-1α as a protective mechanism for cells against hypoxia.

When hypoxia induces HIF-1α signaling in cells, it also set in motion many HIF-1α-independent cascades, wherein important regulators in tissues and cells, like AMP-activated protein kinase/mechanistic target of rapamycin/p70S6 kinase signaling [14], endoplasmic reticulum stress [15], Src [16], extracellular signal-regulated kinase (ERK) [17], nuclear factor kappa B [18], and Akt [19], come into play [20]. While it remains unclear how certain regulators above may contribute to combatting hypoxia, such as Src, many of the regulators has been proven to facilitate cell survival under hypoxia: ERK activation can protect kidney cells from ischemia/hypoxia injury through the MEK/ERK-ABCG2 pathway [21, 22]; enhanced activation of hypoxia and reoxygenation-mediated PI3K/AKT improved the viability of kidney epithelial cells [23]. Although many anti-hypoxic cascades are dependent on HIF-1α, whether HIF-1α is the most important pathway for targeting AKI during hypoxia has not been determined.

Yes-associated protein (YAP) is a well-known transcriptional factor for cell survival [24, 25]. The nucleocytoplasmic distribution of YAP is a key determinant of its activity and function. Cytoplasmic YAP is usually functionless, while nuclear YAP has the transcriptional ability to regulate gene expression. Before YAP imports into nucleus, it has to be dephosphorylated. In a nutshell, the exertion of its effects is subject to both its translocation into nucleus, and its de-phosphorylation. Its interactions within the Hippo pathway, one of the most researched subject, clearly doesn’t meet the above two requisites, as YAP’s interaction with other core components in the pathway, such as mammalian sterile 20-like kinase 1/2 (MST1/2), large tumor suppressor 1/2 (LATS1/2), adapter protein Salvador homolog 1 (SAV1), and MOB kinase activators (MOB1A and MOB1B) [26], not only promotes the phosphorylation of YAP at serine 127 (Ser127), but also keeps YAP in the cytoplasm, and inhibits YAP/TAZ transcription co-activators [27, 28]. AKT also phosphorylates YAP at Ser127, which facilitates the binding of YAP to 14-3-3 and retains YAP in the cytoplasm [29]. By contrast, ERK2 phosphorylates 14-3-3ζ at Ser37, which induces the nuclear localization of YAP under hypoxic conditions [30]. In fact, the inhibition of ERK1/2 is often associated with the decrease of YAP protein expression [31]. It’s worth pointing out that YAP transcriptional activity can either promote tissue fibrosis or regeneration for different types of renal cells [32]. While YAP activation can excite myofibroblast proliferation and ECM production, leading to renal fibrosis [33], YAP activation via the EGFR-PI3K-Akt pathway promotes renal epithelial cell recovery from fibrosis in an AKI mouse model [34].

Although YAP can shuttle between the nucleus and the cytoplasm, it lacks canonical nuclear localization signals (NLS) [35]. Many studies have demonstrated the possible mechanisms in controlling YAP nuclear entry [36–40]. However, it remains unclear whether there is a direct carrier that enables YAP nuclear translocation. This means the potential carrier of YAP nuclear localization has yet to be identified. As hypoxia upregulates several signaling pathways that regulate the activity and localization of YAP, we propose that hypoxia-induced HIF-1α controls YAP localization and mediates its function during hypoxic conditions. For the present, we only know that both HIF-1α and YAP have respective beneficial effects in the early stages of AKI, and that YAP can stabilize HIF-1α [41], while HIF-1α can also promote YAP expression and activation [42, 43]. That’s to say, it has yet to be clarified whether the interaction of HIF-1α and YAP has protective effects in the early stages during hypoxic insults.

We used pharmacological and genetic approaches to address the above question and discovered that not only do HIF-1α and YAP have protein-protein interactions, but also colocalize in nucleus. We also examined DNA damage and apoptosis under hypoxic conditions in the presence of HIF-1α and YAP interactions, and came to see how the interaction between HIF-1α and YAP prevents hypoxia-induced cell injury in renal epithelial cells. The results of this research will lead to an increased understanding of how hypoxic insults affect renal epithelial cells and demonstrate a possible direction for treating hypoxia-induced renal injury such as AKI in the future.

## Results

### YAP and HIF-1α co-localize in nucleus under CoCl_2_-mediated hypoxia

**Fig. 1:**
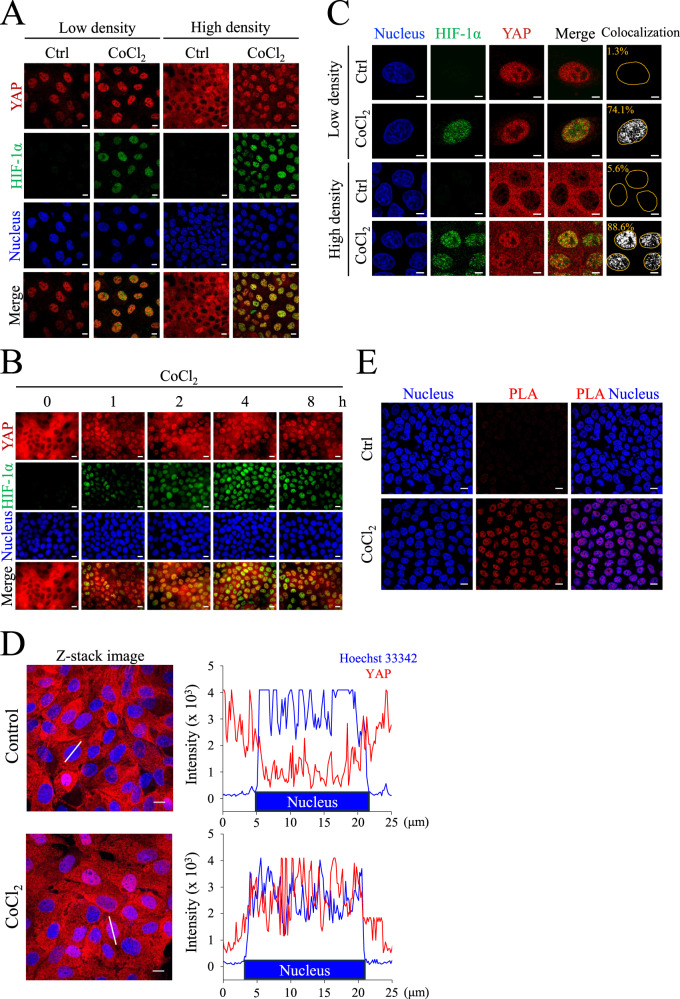
Localization and activation of YAP at low and high cell densities under CoCl_2_ treatment. **A** MDCK cells at low density (5 × 10^4^) and high density (3 × 10^5^) were treated with or without 400 μM CoCl_2_ for 8 h. Immunofluorescent images of YAP (red) and HIF-1α (green) were taken using confocal microscope. Scale bar: 10 μm. **B** MDCK cells at high density were treated with 400 μM CoCl_2_ for 0, 1, 2, 4, and 8 h. Immunofluorescent images of YAP (red) and HIF-1α (green) were obtained using confocal microscope. Scale bar: 10 μm. **C** Low- and high-density MDCK cells were in the absence or presence of 400 μM CoCl_2_ for 8 h. Immunofluorescence confocal images of YAP (red) and HIF-1α (green) were analyzed using FluoView FV10-ASW (Olympus). The colocalization of YAP and HIF-1α were shown in white. The percentage of YAP and HIF-1α colocalization are indicated in the images (orange character). Scale bar: 5 μm. **D** High-density MDCK cells were seeded in the collagen gel, and subjected to control or CoCl_2_ for 8 h. Representative Z-stack images of YAP (red) and nuclei (blue) were analyzed using FluoView FV10-ASW (Olympus). The right panel showed the fluorescence intensity profiles of solid lines in the Z-stack images. Scale bar: 10 μm. **E** MDCK cells at high density were treated with or without 400 μM CoCl_2_ for 8 h. The in situ PLA determined the physical interactions between HIF-1α and YAP. Nuclei shown in blue were labeled by Hoechst 33342. Scale bar: 10 μm.

To examine the localization of YAP under normoxia and hypoxia in Madin-Darby canine kidney (MDCK) cells, we treated the cells with the hypoxia mimetic compound CoCl_2_ for 8 h at low and high cell densities. Immunofluorescence staining was performed to observe the localization of YAP. Compared to the normoxic group, images showed that YAP translocated to the nucleus under CoCl_2_-mediated hypoxia at a high cell density (Fig. 1A). The quantification data also showed a significant increase in YAP nuclear localization in the CoCl_2_-mediated hypoxic group compared to that in the normoxic group at a high cell density (Fig. S1A). Next, we verified whether YAP nuclear translocation was induced by CoCl_2_-mediated hypoxia using immunofluorescence staining. Images demonstrated that nuclear translocation of HIF-1α and YAP was increased in a time-dependent manner under CoCl_2_ incubation (Fig. 1B). Analysis of HIF-1α and YAP nuclear fluorescence intensities showed that CoCl_2_-mediated hypoxia enhanced the nuclear accumulation of HIF-1α and YAP in the same manner (Fig. S1B). Immunofluorescence images also illustrated an increase in the colocalization ratio of HIF-1α and YAP under CoCl_2_-mediated hypoxia at low and high cell densities (Fig. 1C). Although YAP and TAZ are paralogous transcriptional regulators that are involved in the hippo pathway, hypoxic conditions cannot induce TAZ nuclear translocation under high cell density (Fig. S1C). In addition, the co-translocation of HIF-1α and YAP was also found in a human renal epithelial cell line, HK-2 cells (Fig. S2A, B). Considering that YAP is a mechanical sensitive transcriptional factor, the extracellular environment may affect the localization of YAP. To better understand hypoxia-induced YAP translocation under physiological conditions, we seeded MDCK cells in the 3D collagen gel. Results showed that CoCl_2_-induced nuclear YAP localization can also be found under high-density collagen gel culture (Fig. 1D). These results suggest that HIF-1α and YAP are co-expressed in the nucleus under hypoxic conditions.

### CoCl_2_-mediated hypoxia increases the physical interaction of YAP and HIF-1α in the nucleus

**Fig. 2:**
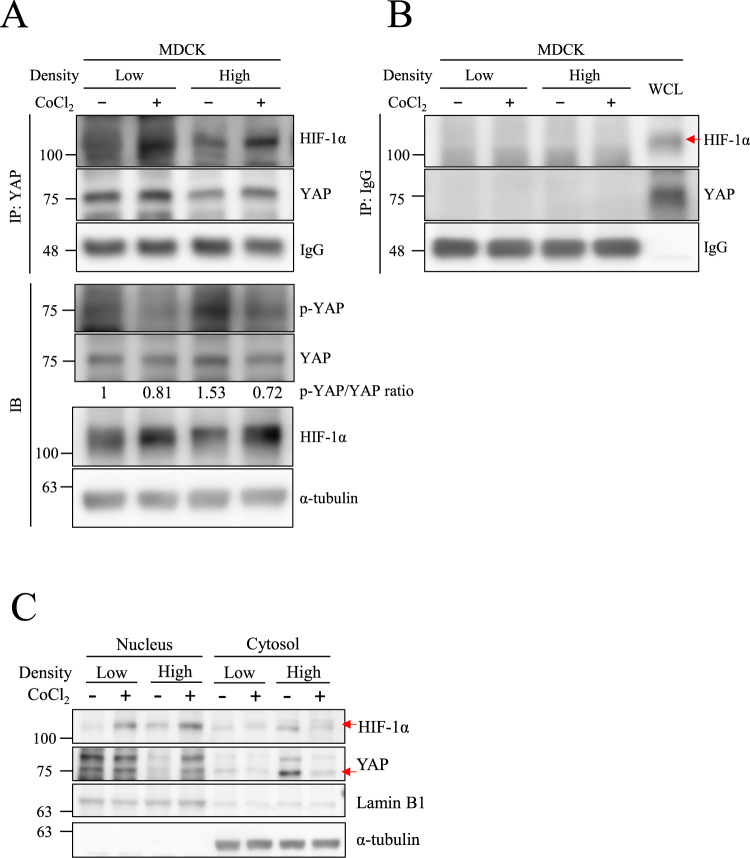
CoCl_2_ increases nuclear HIF-1α and YAP protein-protein interaction under high cell density. **A** MDCK cells at low and high density were incubated with or without 400 μM CoCl_2_ for 8 h. The immunoprecipitations were performed and analyzed by Western blotting with anti-YAP antibodies. IgG was used as a loading control. Total cell lysates were subjected to Western blotting with indicated antibodies, and α-tubulin was used as a loading control. **B** The negative control for the co-immunoprecipitation experiment was performed using an anti-IgG antibody. Whole cell lysate (WCL) is the positive control for anti-YAP and anti-HIF-1α antibodies. IgG was used as a loading control of immunoprecipitation samples. **C** MDCK cells at low and high density were treated with or without 400 μM CoCl_2_ for 8 h. Both nuclear and cytosol extracts were analyzed by Western blotting. Lamin B1 and α-tubulin were used as a loading control for the nuclear and cytosolic proteins.

Some studies have reported an interaction between HIF-1α and YAP. Hence, we proposed that YAP nuclear translocation was transported by HIF-1α. Proximity ligation assay (PLA) was conducted to evaluate the physical interaction of HIF-1α and YAP under hypoxic conditions. Images showed that PLA signals were significantly increased in the CoCl_2_-treated group, indicating the physical interactions of HIF-1α and YAP were induced in the nucleus under hypoxic conditions (Fig. 1E). Immunoprecipitation also revealed that CoCl_2_ increased HIF-1α and YAP interactions compared to normoxia under both low- and high-cell density conditions (Figs. 2A, S3). Lysates that immunoprecipitated with anti-IgG antibody showed no signal in each group, excluding the false positive signals in Fig. 2A (Fig. 2B). Western blotting also showed a decrease in phospho-YAP (Ser127) levels, an inhibitory phosphorylation site, under CoCl_2_-mediated hypoxia compared to normoxia at a high cell density (Fig. 2A). Moreover, the CoCl_2_ induced the expression levels of HIF-1α and YAP in the nuclear fraction under high density as well. (Fig. 2C). As YAP is a transcriptional factor, we next use PCR to verify the gene expression of its downstream gene *cyr61* under hypoxia. Results revealed that hypoxia increased *cyr61* expressions in MDCK-Parental cells but not in MDCK-shHIF-1α cells (Fig. S4). These results indicate that the interaction of HIF-1α and YAP facilitates YAP nuclear translocation under hypoxia-like treatment in high-cell density conditions.

### CoCl_2_-mediated hypoxia inhibits Hippo pathway and promotes YAP nuclear translocation

**Fig. 3:**
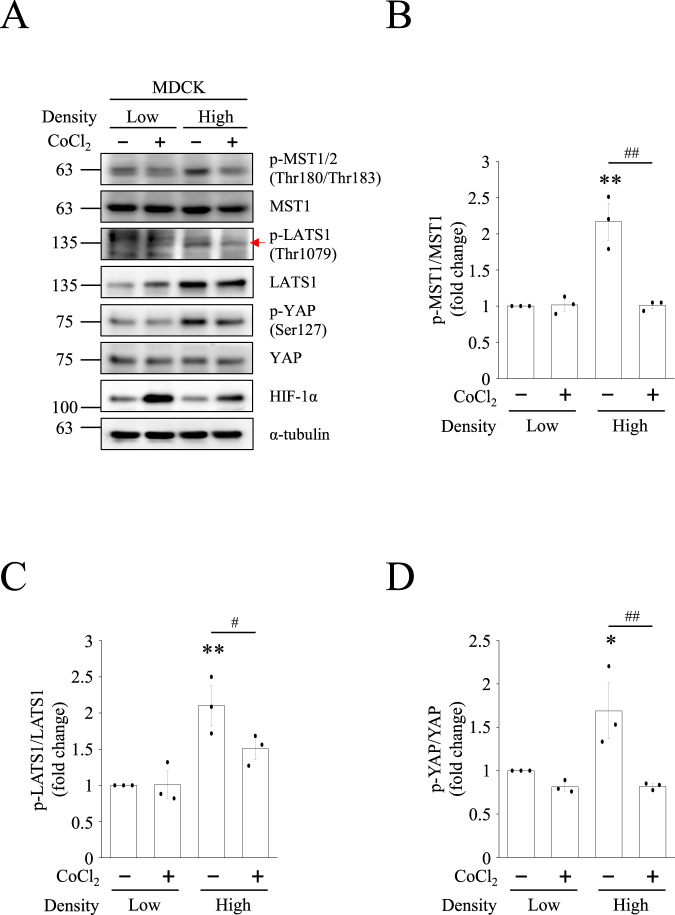
CoCl_2_ decreases Hippo pathway under high cell density conditions. **A** MDCK cells at low and high density were treated with or without 400 μM CoCl_2_ for 8 h. Protein levels of Hippo pathway in each group were determined by Western blotting. The quantification of (**B**) phospho-MST1/MST1, (**C**) phospho-LATS1/LATS1, and (**D**) phospho-YAP/YAP were presented as mean ± SEM. *p < 0.05 and **p < 0.01 vs. control group in low-cell density conditions. ^#^*p* < 0.05 and ^##^*p* < 0.01 vs. control group in high-cell density conditions.

YAP is a major effector of the Hippo pathway. To investigate whether this Hippo pathway is involved in hypoxia-induced nuclear translocation of YAP, we verified the expression levels of Hippo pathway proteins under CoCl_2_-mediated hypoxia. The results showed that high cell densities promoted the activation of MST1/2 and LATS1 under normoxic conditions. However, CoCl_2_ inhibited MST1/2 and LATS1 phosphorylation at high cell densities (Fig. 3A). The quantified data also showed a significant decrease in the active phospho-MST1 (Thr180/Thr183)/MST1 and active phospho-LATS1 (Thr1079)/LATS1 ratio under CoCl_2_-mediated hypoxia (Fig. 3B, C). In contrast, CoCl_2_-mediated hypoxia suppressed inhibitory phospho-YAP (Ser127) levels to a greater extent than in the normoxic group. The quantitative data showed a significant decrease in the phospho-YAP (Ser127)/YAP ratio under CoCl_2_-mediated hypoxia (Fig. 3D). In consistent with the above results in MDCK cells, HK-2 cells also showed the same pattern of the MST1, LATS1, and YAP phosphorylation (Fig. S2C). These data indicate that hypoxia-like conditions inhibit the Hippo pathway and promote YAP nuclear localization.

### YAP nuclear translocation depends on AKT pathway

**Fig. 4:**
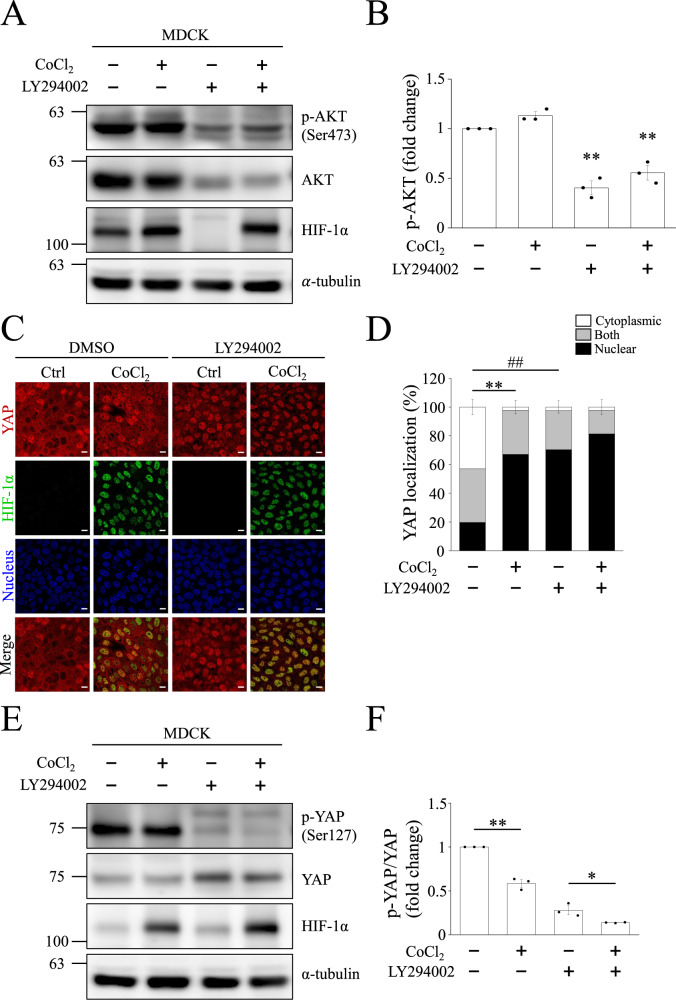
AKT inhibitor decreases YAP phosphorylation at serine 127 under high-cell density conditions. High density MDCK cells were treated with or without 10 μM LY294002 for 24 h, followed by control or 400 μM CoCl_2_ treatment for 8 h. **A** Representative blots of phospho-AKT, AKT, and HIF-1α were shown and α-tubulin was used as a loading control. **B** Quantification of phospho-AKT in (**A**). Bars represent the mean ± SEM. **p < 0.01 vs. CoCl_2_-only group. **C**, **D** Immunofluorescence images of YAP (red) and HIF-1α (green) obtained using confocal microscope. Scale bar: 10 μm. Proportion of YAP localization in cytoplasm, nucleus, or both after LY294002 treatment was quantified in (**D**). Bars represent the mean ± SEM. **p < 0.01, ^##^*p* < 0.01. Representative Western blots are shown in (**E**), and fold change in phospho-YAP and YAP is quantified in (**F**). Bars represent the mean ± SEM. *p < 0.05 and **p < 0.01.

Besides the HIF-1α-dependent pathway, non-HIF-1α dependent pathways, including AKT, ERK, and Src, also participate in hypoxia-induced YAP nuclear translocation. Therefore, we first used the AKT inhibitor, LY294002, to evaluate whether AKT is involved in hypoxia-induced YAP nuclear translocation in MDCK-Parental cells. Western blots showed that active phospho-AKT (Ser473) was not enhanced by CoCl_2_-mediated hypoxia at a high cell density. However, LY294002 suppressed phospho-AKT (Ser473) levels under normoxic and CoCl_2_-mediated hypoxia conditions (Fig. 4A, B). These results can also be found in MDCK-shHIF-1α cells (Fig. S6C). We evaluated YAP localization in MDCK-Parental cells following LY294002 treatment using immunofluorescence staining. The images revealed that YAP was translocated to the nucleus even under normoxia in the presence of LY294002 (Fig. 4C). Quantification of YAP localization also indicated a significant increase in nuclear YAP in the LY294002-only group (Fig. 4D). MDCK-shHIF-1α cells also showed the same phenomena as found in the parental cells (Fig. S6A, B). Since YAP localization is determined by its phosphorylation state, we investigated whether YAP phosphorylation varied after CoCl_2_ or LY294002 treatment. We found that phospho-YAP (Ser127) was downregulated by LY294002 treatment (Fig. 4E). The quantified phospho-YAP (Ser127)/YAP ratio further showed a significant decrease in the LY294002 + CoCl_2_ group compared to the LY294002-only group. CoCl_2_-mediated hypoxia also inhibited the phospho-YAP (Ser127)/YAP ratio compared to normoxia (Fig. 4F). These data demonstrate that the activation of AKT induces the phosphorylation of YAP at Ser127 and prevented YAP nuclear translocation at a high cell density. However, AKT activation did not affect YAP dephosphorylation and nuclear translocation by CoCl_2_.

Next, we examined whether Src and ERK participated in the hypoxia-induced nuclear translocation of YAP. Western blots showed that Src phosphorylation did not change at high cell densities under CoCl_2_-mediated hypoxia compared to that under normoxia (Fig. S5A). While the ratio of active phospho-Src (Tyr416)/Src was significantly decreased in the dasatinib-treated groups, no significant differences were observed between the dasatinib-only and dasatinib + CoCl_2_ groups (Fig. S5B). In addition, the active phospho-ERK2 (Thr202/Tyr204)/ERK2 ratio was induced by CoCl_2_-mediated hypoxia, whereas the ERK inhibitors SCH77298 and U0126 prevented the elevation of the phospho-ERK2 (Thr202/Tyr204)/ERK2 ratio in normoxia or CoCl_2_-mediated hypoxia (Fig. S5A, C). Moreover, the phospho-YAP (Ser127)/YAP ratio was reduced in all CoCl_2_-mediated hypoxia groups (Fig. S5D). Immunofluorescence images showed that YAP nuclear translocation was not significantly different between the CoCl_2_-only group and the dasatinib, SCH77298, or U0126 co-treated with CoCl_2_ groups (Fig. S5E, F). These data suggest that the Src and ERK pathways are not involved in YAP nuclear translocation under hypoxia-like conditions.

### HIF-1α is crucial for YAP nuclear translocation under normoxia or CoCl_2_-induced hypoxic conditions

**Fig. 5:**
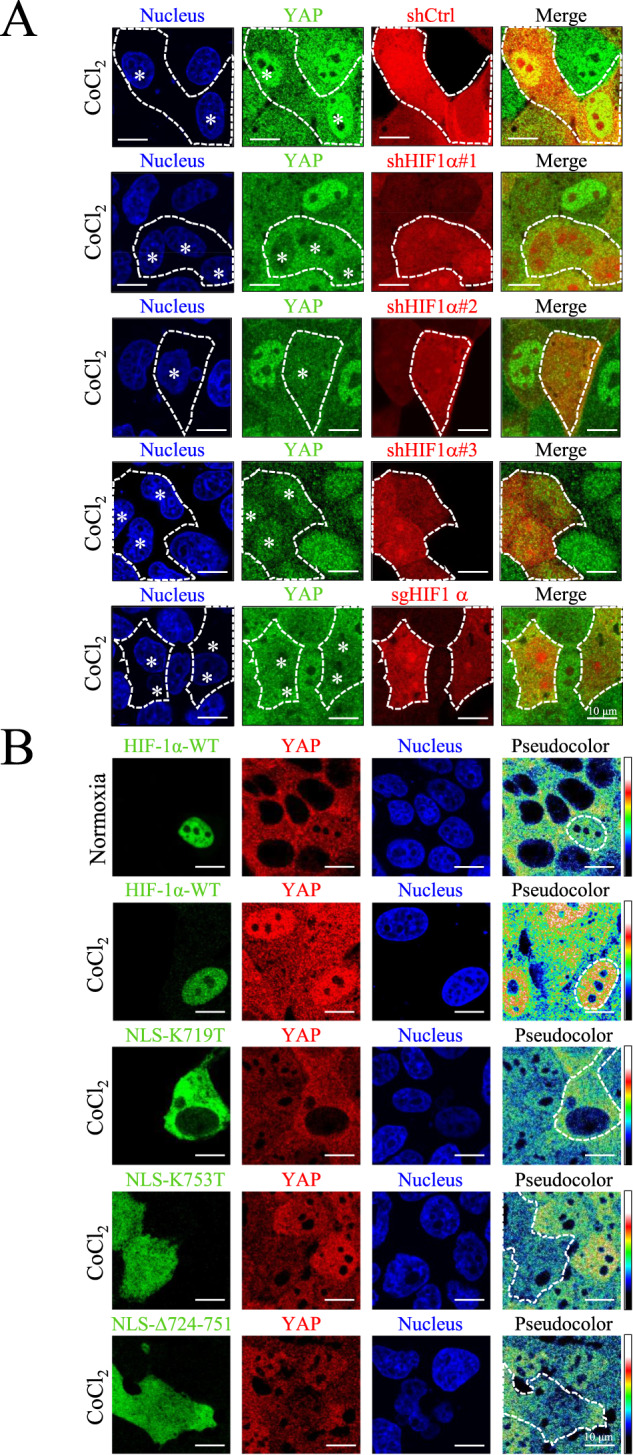
HIF-1α synchronizes YAP nuclear translocation in HIF-1α knockdown, knockout and overexpressed cells. **A** High-density MDCK-shCtrl, MDCK-shHIF-1α #1, #2, #3, and MDCK-sgHIF-1α cells were treated with 400 μM CoCl_2_ for 8 h. Immunofluorescence images of YAP (green), shCtrl cells (red), shHIF-1α cells (red), and sgHIF-1α cells (red) were obtained using a confocal microscope. White dashed lines indicate the border of shCtrl, shHIF-1α and sgHIF-1α cells. White stars indicate the nuclei of shCtrl, shHIF-1α and sgHIF-1α cells. Scale bar: 10 μm. **B** Cells were either transient transfected with HIF-1α-WT-GFP (in MDCK-parental cells) or HIF-1α-NLS mutants: HIF-1α-NLS-K719T-GFP, HIF-1α-NLS-K753T-GFP, and HIF-1α-NLS-Δ724-751-GFP (in MDCK-shHIF-1α cells). The transfected MDCK-parental cells were then subjected to normoxia and 400 μM CoCl_2_, and the transfected MDCK-shHIF-1α cells were subjected to 400 μM CoCl_2_, respectively. After 8 h, images of HIF-1α (green), YAP (red and pseudo-color), and nuclei (blue) were captured using confocal microscope. Pseudocolor images show expression pattern of YAP. White dashed lines indicate the border of exogenous HIF-1α variants. Scale bar: 10 μm.

To verify whether HIF-1α is necessary for YAP nuclear localization under CoCl_2_-mediated hypoxia, MDCK cells with vector control (shCtrl), HIF-1α knockdown (shHIF-1α#1, #2, and #3), and knockout (sgHIF-1α) were used. Confocal microscopy images revealed that depletion of HIF-1α prevented YAP nuclear localization under CoCl_2_-mediated hypoxia (Fig. 5A). Because YAP has no NLS, we assumed that YAP nuclear translocation was transported by HIF-1α. We transfected cells with HIF-1α-WT or HIF-1α-NLS mutants (NLS-K719T, NLS-K753, and NLS-Δ724-751). Even under normoxia, YAP could be transported in the nucleus of HIF-1α-WT overexpressing cells. In contrast to HIF-1α-WT overexpressing cells, YAP nuclear translocation failed and retained in cytoplasmic in NLS-K719T, NLS-K753, and NLS-Δ724-751 cells under CoCl_2_-mediated hypoxia (Fig. 5B). These results suggest that NLS is important for HIF-1α-mediated YAP nuclear translocation.

### MDCK-shHIF-1α cells have more DNA damage and apoptosis levels than parental cells under hypoxic conditions

**Fig. 6:**
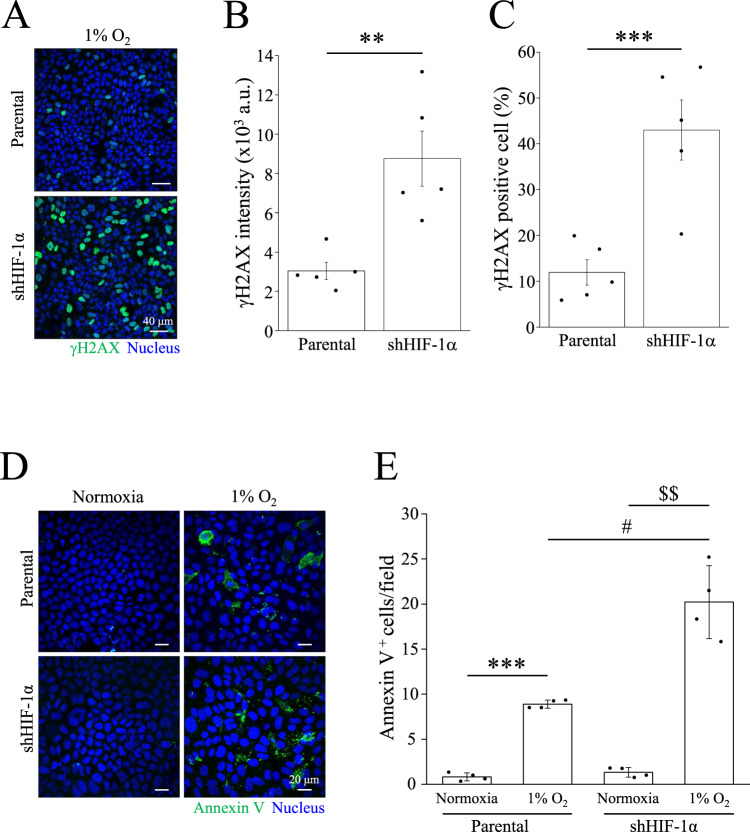
Increase of γH2AX and Annexin V in MDCK-shHIF-1α cells under hypoxic conditions. **A** High density cells were subjected to 1% O_2_ for 24 h, followed by immunostaining with anti-γH2AX antibody. Scale bar: 40 μm. **B**, **C** γH2AX intensity and γH2AX positive cells were analyzed. Bars were presented as mean ± SEM. *p < 0.05, **p < 0.01. **D** High density cells were subjected to normoxia or 1% O_2_ for 36 h, followed by Annexin V staining. Scale bar: 20 μm. **E** The number of Annexin V positive cells were analyzed and presented as mean ± SEM. ***p < 0.001 vs. normoxia in parental cells, ^$$^*p* < 0.01 vs. normoxia in shHIF-1α cells, and ^#^*p* < 0.05 vs. hypoxia in parental cells. All immunofluorescence images were obtained using confocal microscope.

Hypoxia can cause dysfunction in DNA repair. We subjected MDCK-parental and MDCK-shHIF-1α cells to 1% O_2_ for 48 h. Hoechst 33342 staining indicated that MDCK-shHIF-1α cells displayed severe DNA condensation and nuclear deformation upon exposure to 1% O_2_ (Fig. S7). To further examine the causes of the DNA breaks, the cells were stained with the DNA damage marker γH2AX. Immunofluorescence images showed a higher expression level of γH2AX was found in MDCK-shHIF-1α cells compared to parental cells after treatment with 1% O_2_ (Fig. 6A). Quantitative data also revealed significant increases in the intensity of γH2AX and the percentages of γH2AX-positive cells under 1% O_2_ (Fig. 6B, C). In addition to 1% O_2_, CoCl_2_-mediated hypoxia increased the γH2AX intensity in MDCK-shHIF-1α cells (Fig. S8A, B). Furthermore, the number of apoptotic cells was also elevated in MDCK-shHIF-1α cells under 1% O_2_ (Fig. 6D). Quantitative data indicated a significant increase in Annexin V-positive cells in MDCK-shHIF-1α cells compared to that in parental cells (Fig. 6E). HK-2 cells with shHIF-1α transient transfection also showed an increase of γH2AX and Annexin V-positive signal under hypoxia (Fig. S2D, E). These data demonstrate that HIF-1α-induced YAP nuclear localization may prevent cells from DNA damage and apoptosis under hypoxic conditions.

### YAP is necessary for DNA protection and cell survival under hypoxic conditions

**Fig. 7:**
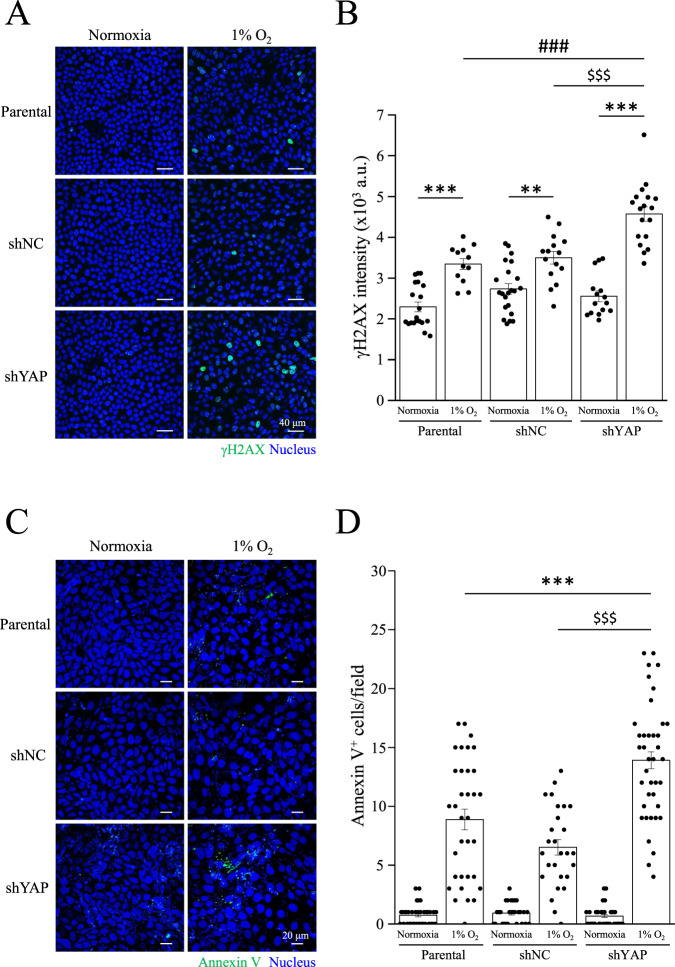
Protective effects of YAP under hypoxic conditions. **A**, **B** MDCK-parental, MDCK-shNC, and MDCK-shYAP cells were subjected to normoxia or 1% O_2_ for 24 h, followed by immunostaining with anti-γH2AX antibody. Scale bar: 40 μm. γH2AX intensity was analyzed and presented as mean ± SEM in (**B**). ***p < 0.001, **p < 0.01, ^$$$^*p* < 0.001, and ^###^*p* < 0.001. **C**, **D** MDCK-parental, MDCK-shNC, and MDCK-shYAP cells were subjected to normoxia or 1% O_2_ for 36 h, followed by Annexin V staining. Scale bar: 20 μm. **D** Annexin V positive cell number was analyzed and presented as mean ± SEM. ***p < 0.001 vs. 1% O_2_ in parental cells, ^$$$^*p* < 0.001 vs. 1% O_2_ in shNC cells.

**Fig. 8:**
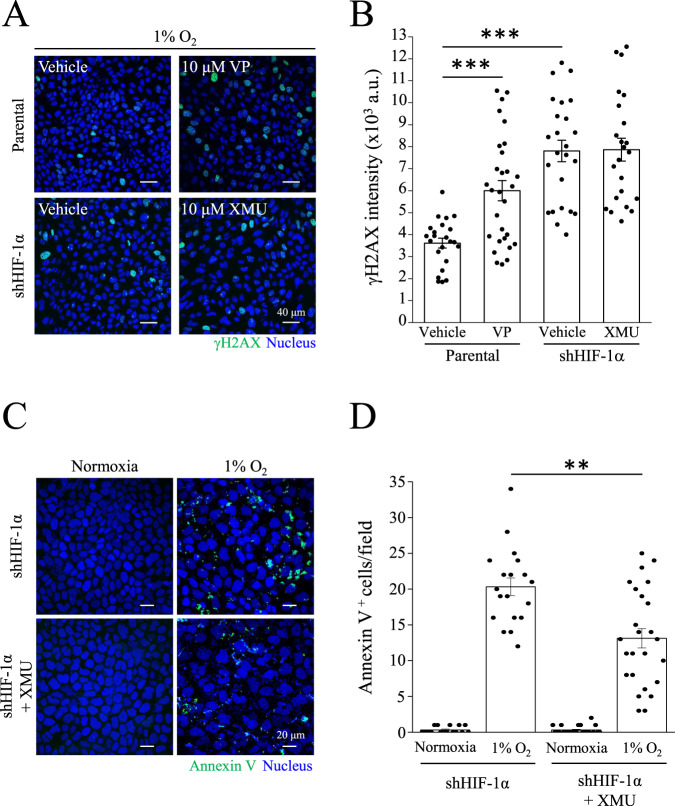
Rescue effects of XMU-MP-1 in shHIF-1α cells. **A**, **B** MDCK-parental cells were treated with vehicle (Veh) or 10 μM verteporfin (VP) under 1% O_2_. MDCK-shHIF-1α cells were treated with Veh or 10 μM XMU-MP-1 (XMU) under 1% O_2_. After 24 h, MDCK-parental and MDCK-shHIF-1α cells were immunostaining with anti-γH2AX antibody. Scale bar: 40 μm. **B** γH2AX intensity was analyzed and presented as mean ± SEM. ***p < 0.001. **C**, **D** MDCK-shHIF-1α cells were treated with Veh or XMU under normoxia or 1% O_2_ condition for 36 h, followed by Annexin V staining. Scale bar: 20 μm. **D** Annexin V positive cell number was analyzed and presented as mean ± SEM. **p < 0.01. All images were obtained using confocal microscope.

Next, we generated the MDCK-shNC and MDCK-shYAP cell lines to further understand the role of YAP in nuclear translocation under hypoxic conditions. As expected, YAP-knockdown cells had higher γH2AX intensity compared to MDCK-parental and MDCK-shNC cells under 1% O_2_ (Fig. 7A, B). The number of apoptotic cells also increased in MDCK-shYAP cells, but not in MDCK-parental or MDCK-shNC cells (Fig. 7C, D). Next, we verified whether YAP activation protected cells from DNA damage and apoptosis. MDCK-parental cells were treated with verteporfin (VP), a YAP inhibitor. In contrast, we treated MDCK-shHIF-1α cells with the YAP activator XMU-MP-1 (XMU). Results showed that VP upregulated γH2AX levels in MDCK-parental cells under 1% O_2_ (Fig. 8A, B). Although XMU did not prevent γH2AX levels in MDCK-shHIF-1α cells, XMU reduced the number of apoptotic cells under 1% O_2_ (Fig. 8C, D). A constitutively active form of YAP with a Ser127 to alanine mutant (S127A) remains in the nucleus and is transcriptionally active. Under 1% O_2_ condition, YAP-S127A transfected cells reduced γH2AX levels compared to YAP-WT transfected cells in MDCK-shHIF-1α cell lines (Fig. S9A, B). Moreover, hypoxia enhanced the expression of another DNA damage marker, Rad51, in MDCK-shHIF-1α and MDCK-shYAP cells (Fig. S10A). Increases of cleaved-PARP and cleaved-caspase 3 were also identified in MDCK-shHIF-1α and MDCK-shYAP cells under hypoxia (Fig. S10B). These data indicate that YAP activity is critical for preventing DNA damage and apoptosis under hypoxic conditions.

## Discussion

The main objective of the current study is to investigate the interactions between HIF-1α and YAP under hypoxic conditions. We found in spite of high cell confluency, YAP nuclear translocation still transpired in MDCK and HK-2 cells treated with a low level of O_2_ (1%) or CoCl_2_-mediated hypoxia (Figs. 1, S2). Both results from PLA and immunoprecipitations confirmed the physical interaction between HIF-1α and YAP under hypoxia (Figs. 1, 2). Using genetic modifications, we validated that the NLS motif of HIF-1α is required for HIF-1α-induced nuclear translocation of YAP under hypoxia (Fig. 5). The results from ɤH2AX and AnnexinV staining demonstrated that the interaction of HIF-1α and YAP reduced DNA damage and apoptosis induced by hypoxia (Figs. 6, 7, 8). Taken together, we conclude that HIF-1α serves as a carrier through which YAP translocates into nucleus of renal epithelial cells, and once imported, nuclear YAP exerts a preventive effect against DNA damage and apoptosis under hypoxic conditions.

Contact inhibition usually activates the Hippo pathway at high cell densities, which phosphorylates YAP at Ser127 and inhibits the nuclear translocation of YAP [44, 45]. This rationale accounts for why most research in the past found high confluency inhibitory for YAP nuclear translocation. By contrast, even though the MDCK and HK-2 cells were highly confluent in our study, treating them with CoCl_2_-mediated hypoxia led to the suppression of the Hippo pathway, consequently YAP dephosphorylation at Ser127 and nuclear translocation (Figs. 1A, S1A, 3A). Judging from the results, the Hippo pathway is involved in hypoxia-induced nuclear localization of YAP in an inhibitory way. Our findings are consistent with a previous study published by Lin et al., who pointed out that hypoxia downregulates the protein and mRNA levels of LATS1 by the direct binding of HIF-1α to the hypoxia response element of LATS1. Their study attests YAP nuclear translocation in eutopic stromal cells under hypoxia and the increase of its transcriptional activity [46]. The authors went on to conclude that hypoxia inhibits Hippo pathway through transcription, translation, and post-modification of LATS1, which contributes to YAP activation and nuclear translocation.

Since the phosphorylation of YAP, which is inhibitory for its activity, is mainly controlled by the Hippo pathway, and there has been a significant increase in the interaction between and colocalization of HIF-1α and YAP under hypoxia, we inferred that YAP translocation may occur via two steps: (1) the phosphorylation of YAP has to be attenuated, and (2) HIF-1α serves as a carrier for YAP to enable co-transportation. The first step is necessary because YAP phosphorylated by Hippo pathway at Ser127 is typically sequestered by 14-3-3 and kept in the cytoplasm; only after dephosphorylation is YAP released from 14-3-3 and can therefore interact with HIF-1α freely under hypoxic conditions. The co-translocation of HIF-1α and YAP indicates that HIF-1α is a potential carrier for the nuclear transport of YAP. Further experiments are required to examine whether YAP dephosphorylation is necessary for the interaction of YAP and HIF-1α.

Although nuclear-cytoplasmic shuttling of YAP has been reported in many studies, none have identified the specific NLS motif for YAP under hypoxia. When mechanical stimulation is applied to cells and nuclear pores deform as a result, an increase in the rate of YAP nuclear translocation can also be observed [36]. This is because YAP is a relatively larger protein (>50 kDa) and the deformation of nuclear pores facilitate its translocation. For a protein as large as YAP, it usually takes a transporter to enable the nuclear-cytoplasmic shuttling of YAP and ferry it into the nucleus [47]. One study revealed that ANKHD1 and ANKRD17, which contain both NLS and nuclear export signal, regulate nuclear translocation of YAP by directly binding to YAP [48]. However, the study wasn’t conducted under a hypoxia model. With PLA and immunoprecipitation performed under hypoxic conditions, we have come to attest the interaction between YAP and HIF-1α (Figs. 1E, 2A, S3). With the analysis of confocal microscopy images, we observed a significant increase in the colocalization ratio of YAP and HIF-1α (Fig. 1C). In addition, the nuclear fractions exhibited high expressions of HIF-1α and YAP (Fig. 2C). Moreover, we found YAP retained in the cytosol and failed to transport to the nucleus in a HIF-1α knockout experiment (Fig. 5A). Take the above results and the fact that YAP lacks an NLS motif itself into account, we further investigated whether the NLS motif of HIF-1α holds the key to YAP nuclear transportation with three NLS mutant HIF-1α variants as the subjects, and verified that the three failed to induce YAP nuclear transport under CoCl_2_-mediated hypoxia (Fig. 5). It’s worth noting that in cells genetically engineered for HIF-1α-WT upregulation, HIF-1α-WT has been found to promote the nuclear import of YAP, even under normoxic conditions at high cell densities (Fig. 5). These data support the notion that the NLS motif of HIF-1α is the key that enables YAP nuclear transport. The current study is the first to identify the requirement of the HIF-1α NLS motif in promoting the nuclear translocation of YAP under hypoxic conditions. In the research to come, it would be interesting to examine YAP nuclear translocation in relation to homeostasis that seeks to strike a balance between nuclear import and export, as well as investigate if exportins regulating YAP are inhibited under hypoxia.

We also performed experiments to determine the association between YAP nuclear translocation and Src, ERK1/2 as well as AKT. Cell adhesion promotes phospho-Src to inhibit tyrosine phosphorylation of LATS1 and thereby the Hippo pathway [49]. In the present study, Src phosphorylation was found unchanged (Fig. S5), and the phosphorylation of LATS1 at threonine 1079 reduced under CoCl_2_-mediated hypoxia at high cell densities (Fig. 3). Since Src fails to exert an inhibitory effect on the Hippo pathway under hypoxia, we ruled out its participation in YAP translocation under CoCl_2_-mediated hypoxia. Our experiment also told a different story about ERK1/2 and YAP: previous studies have affirmed that ERK2 phosphorylated 14-3-3ζ at serine 37, which induces the nuclear translocation of YAP under hypoxic conditions [30, 50], and that ERK1/2 inhibition downregulates YAP protein levels and Hippo reporter activity [31], implying a positive association between ERK activation and YAP activity; our experiment, by contrast, points out neither inducing ERK1/2 phosphorylation with CoCl_2_-mediated hypoxia nor introducing ERK inhibitors into the experiment makes a difference in the nuclear localization of YAP, which is to say that the ERK pathway is not involved in YAP translocation (Fig. S5). Last but not least, results from our experiment to investigate AKT’s role in YAP nuclear import remain in line with those from previous research: phosphorylated AKT can phosphorylate YAP at Ser127, facilitate its binding to 14-3-3ζ, and sustain YAP in the cytosol [29]. Our data also showed that the AKT inhibitor LY294002 prevented high cell density-induced YAP phosphorylation and induced nuclear accumulation of YAP (Fig. 4). If not for LY294002, AKT would suppress YAP activity by phosphorylating YAP at Ser127 and prevent its nuclear translocation at high cell densities. As a side note, we originally thought hypoxia played a positive role in AKT activation [51], but it’s been observed in our experiment that CoCl_2_-mediated hypoxia did not increase AKT phosphorylation. This inconsistency may result from the fact that CoCl_2_ only induces a single biochemical reaction in cells, namely the inhibition of prolyl hydroxylase domain protein, which maintains the concentration of HIF-1α. Contrarily, what low-oxygen level (1%) can induce is much more than a single biochemical reaction, and factors such as increased ROS production or decreased respiration may all be responsible for the enhancement of AKT activation [52].

In studying the biological function of YAP nuclear translocation, we used MDCK-shHIF-1α cells as the subjects and have proven them more susceptible to hypoxia-induced DNA damage and apoptosis than MDCK-parental cells (Fig. 6). Inferring from previous experiment results, we conclude that lack of HIF-1α would impair nuclear translocation of YAP and consequently the protection against apoptosis and DNA damage. Since YAP plays a key role against apoptosis and DNA damage, we used YAP-knockdown cells in conducting a new experiment under hypoxia, and as expected, MDCK-shYAP cells showed more severe DNA damage and apoptosis than the parental cells (Fig. 7). Previous studies have also reported that YAP controls cell cycle, mitosis, and DNA repair, as well as potentiates the transcription of proto-oncogenes and glycolysis. These cellular processes contribute to cell survival and proliferation, once again supporting the idea that YAP and its nuclear translocation have a protective effect over cells against extreme conditions, including hypoxia. To further confirm whether the activity of YAP is critical for preventing DNA damage and apoptosis, we treated MDCK-shHIF-1α cells with the YAP activator XMU under hypoxia. XMU prevents YAP phosphorylation by inhibiting the upstream kinases MST1/2 and LATS1/2. Although results showed that treating MDCK-shHIF-1α cells with XMU does not contribute to preventing DNA damage, it does reduce hypoxia-induced apoptosis (Fig. 8). Results from this experiment imply that YAP activation helps cells develop a high tolerance to DNA damage and protect cells against apoptosis. In another experiment where we used MDCK-shHIF-1α cells with constitutively active YAP-S127A as our subjects, we discovered that hypoxia-induced DNA damage was mitigated (Fig. S9). MDCK-shHIF-1α cells treated with XMU and YAP-S127A differ in their DNA-protecting and anti-apoptotic ability, maybe because XMU activates YAP through downregulating its upstream gene expression in the Hippo pathway, which may inhibit more than one phosphorylation site in YAP, while YAP-S127A works on one phosphorylation site only, Ser127. After proving YAP’s importance in protecting DNA and cells, we also conducted PCR assay on both MDCK-parental cells and MDCK-shHIF-1α cells and found the former showed much more YAP downstream gene expression than the latter (Fig. S4), indicating the protective effect of YAP comes from its transcriptional activity triggered by HIF-1α under hypoxia. In terms of how other YAP phosphorylation sites may affect its translocation, further studies need to be conducted.

In conclusion, the present study is the first to clarify that HIF-1α is a crucial pathway for nuclear translocation of YAP at a high cell density under hypoxic conditions. Moreover, we have proven nuclear translocation and activation of YAP contribute to cell protection under hypoxic conditions. This mechanism may represent a possible remedy for the early stages of IRI-induced AKI. YAP activators can be applied to early-stage AKI patients to prevent severe renal cell death. On the other hand, in the prevention of renal cell damage, YAP activators can also be used as a pretreatment for patients likely to experience hypoxia, such as patients scheduled for a transplantation. Overall, the present study provides insights into YAP as a potential target for the treatment of hypoxia-induced diseases.

## Materials and methods

### Cell culture

MDCK cells were cultured in high-glucose Dulbecco’s modified Eagle’s medium (DMEM; #CC103-0500; Simply Biologics, Taichung, Taiwan) supplemented with 10% fetal bovine serum (FBS; #10437-028; Gibco, Billings, MT, USA) and 1% penicillin/streptomycin (#CC502-0100; Simply Biologics). Cells were all incubated in a humidified incubator at 37 °C and 5% CO_2_.

### Chemicals and reagents

The ERK inhibitor U0126 and the Src inhibitor dasatinib were purchased from Tocris Bioscience (Minneapolis, MN, USA). The ERK inhibitor SCH772984, AKT inhibitor LY294002, YAP inhibitor verteporfin, and YAP activator XMU-MP-1, were purchased from MedChemExpress (Monmouth Junction, NJ, USA). Hypoxia-mimetic CoCl_2_ was purchased from Sigma-Aldrich (St. Louis, MO, USA).

### Lentiviral infection

To generate MDCK-shHIF-1α and MDCK-sgHIF-1α cells, lentiviral vectors encoding shHIF-1α-RFP, sgHIF-1α-RFP, or vector control were introduced to MDCK cells in the presence of polybrene (8 mg/mL) at an MOI of 2 or 6 for 24 h. The lentivirus-containing culture medium was removed and replaced with fresh medium containing 10% FBS and 1% penicillin/streptomycin. The cells were then amplified and selected at least three times using flow cytometry to obtain 99% of the RFP^+^ cell population. shHIF-1α-RFP (#1-#3), sgHIF-1α-RFP, and vector control were purchased from Taiwan Academia Sinica (Taiwan). The target RNAi sequences were as follows: shHIF-1α#1, GTGATGAAAGAATTACCGAAT; shHIF-1α#2, CCGCTGGAGACACAATCATAT; shHIF-1α#3, CGGCGAAGTAAAGAATCTGAA; and sgHIF-1α, TCCGCTCCCCTCGTCGCGTG and GAGCCCATTTCCTCCGCCCG.

### Overexpression of HIF-1α variants

HIF-1a has a NLS domain which can help its nuclear translocation. NLS mutation dampens HIF-1α nuclear translocation and sustains HIF-1a in the cytoplasm. To verify whether HIF-1α can carry YAP nuclear translocation, HIF-1α-WT-GFP were transfected to MDCK-parental cells, and HIF-1α-NLS mutants (HIF-1α-NLS-K719T-GFP, HIF-1α-NLS-K753T-GFP, and HIF-1α-NLS-Δ724-751-GFP) were transfected to MDCK-shHIF-1α cells using Lipofectamine 3000 transfection reagent (#L3000001; Invitrogen, Carlsbad, CA, USA). One day after the transient transfection, MDCK-parental cells carrying HIF-1α-WT-GFP were subjected to normoxia. MDCK-shHIF-1α cells carrying HIF-1α-NLS-K719T-GFP, HIF-1α-NLS-K753T-GFP, or HIF-1α-NLS-Δ724-751-GFP were treated with CoCl_2_ for 8 h. Immunofluorescence staining were subsequently applied to the cells.

### YAP knockdown and overexpression

To generate MDCK-shNC and MDCK-shYAP cells, vector control and short hairpin RNA (shRNA)-mediated knockdown of YAP were performed using Lipofectamine 3000 transfection reagent (#L3000001; Invitrogen). Briefly, MDCK cells were transfected with 0.5 μg DNA, 1.5 μL Lipofectamine 3000 reagent, and 1 μL P3000 in serum-free opti-MEM medium (#31985070; Gibco). After 6–8 h of incubation, the medium was replaced with a regular medium. The cells were then amplified and selected at least three times using flow cytometry to obtain 99% of the RFP^+^ cell population. To generate YAP-overexpressing cells, MDCK-shHIF-1α cells were incubated with YAP-WT-GFP or YAP-S127A-GFP plasmids using Lipofectamine 3000 transfection reagent as mentioned above. MDCK-shHIF-1α-YAP-WT and MDCK-shHIF-1α-YAP-S127A cells were then amplified and selected by flowcytometry at least three times to obtain 99% of the RFP^+^/GFP^+^ double-positive cell populations.

### RNA extreaction and RT-PCR

Total RNA was purified using Total RNA isolation kit (#NA017-0100; Simply Biologics) according to the manufacturer’s instructions. The RNA quality was verified by SpectraMax QuickDrop (San Jose, CA, USA) and reverse-transcribed by using iScript cDNA synthesis kit according to the manufacturer’s instructions (#1708891; Bio-Rad, Hercules, CA, USA). cDNA was used as a template for PCR using specific primers for the following genes: *cyr61* (forward: 5’-GGCTGGAATGCAATTTCG-3’; reverse: 5’-TCCCCATTCTGGTAGATTCG-3’); *yap* (forward: 5’-CTCCCAATCCAGTGCCTTCC-3’; reverse: 5’-ACCTGTGTCCATCTCGTCAAC-3’); *gapdh* (forward: 5’-ACGGCACAGTCAAGGCTGAG-3’; reverse: 5’-CAGCATCACCCCATTTGATGTTGG-3’). PCR was carried out at 95 °C for 5 min followed by 30 cycles at 95 °C for 30 s, 44.9 °C (*cyr61*), 53.5 °C (*yap*), or 51.7 °C (*gapdh*) for 40 s, and 72 °C for 30 s, and a final step at 72 °C for 5 min. PCR products were subjected to 2% agarose gel electrophoresis, stained with ethidium bromide, and visualized under UV light.

### Immunofluorescence staining

MDCK cells were seeded in a 3-cm glass-bottom dish (#16235-1S; Alpha Plus, Taoyuan City, Taiwan). After treatment, cells were fixed with 4% paraformaldehyde (#J61899; Alfa Aesar, Haverhill, MA, USA) for 15 min and permeabilized with 0.5% Triton X-100 for 10 min at room temperature. After blocking with CAS-Block (#00-8120; Invitrogen) for 1 h, cells were incubated with primary antibodies at 4 °C overnight. After washing with phosphate-buffered saline for 1 h, the cells were incubated with Hoechst 33342 (#H3570; Invitrogen) and secondary antibodies for 1 h. Excess antibodies were removed and washed before observation. The following primary antibodies were used: HIF-1α (#GTX127309; GeneTex, Irvine, CA, USA), YAP (#H00010413; Abnova, Taipei City, Taiwan), γH2AX (#05-636; Sigma-Aldrich), TAZ (#70148; Cell Signaling Technologies, Danvers, MA, USA), and Rad51 (#ab88572; Abcam, Cambridge, UK). The following secondary antibodies were used: goat anti-rabbit Alexa Fluor 488-conjugated IgG (#ab15007; Abcam), goat anti-mouse Alexa Fluor 488-conjugated IgG (#ab150113; Abcam), goat anti-rabbit Alexa Fluor 594-conjugated IgG (#ab150116; Abcam), goat anti-mouse Alexa Fluor 594-conjugated IgG (#ab150080; Abcam), goat anti-mouse Alexa Fluor 633-conjugated IgG (#A21052; Invitrogen), and goat anti-rabbit Alexa Fluor 633-conjugated IgG (#A21070; Invitrogen). The images were acquired using a confocal microscope (FV3000; Olympus, Tokyo, Japan). Images from the same experiment were captured with the same parameters to perform semi-quantitative analysis.

### Annexin V staining

2.8 × 10^5^ cells were seeded in a 3 cm glass-bottom dish for approximately 16 h followed by 1% O_2_ supply for 36 h. The medium was then replaced with Annexin V-FITC (#K101-100; Biovision, Cambridge, UK)-or Annexin V-AF647 (#E-CK-A254; Elabscience, Houston, TX, USA)-containing buffer. After staining for 1 h in an incubator, the excess Annexin V was removed. The cells were gently washed three times with Annexin V-binding buffer. Images were obtained using a confocal microscope (FV3000; Olympus) immediately after staining.

### Western blotting

Cell lysates were collected using ice-cold radioimmunoprecipitation assay lysis buffer (50 mM Tris-HCl, 150 mM NaCl, 1% NP-40, 0.5% sodium deoxycholate, and 0.1% sodium dodecyl sulfate [SDS], pH 7.4; #50901; Leadgene, Tainan City, Taiwan) containing a cOmplete protease inhibitor cocktail (#4693132001; Roche, Basel, Switzerland), 1 mM PMSF, 1 mM Na_3_VO_4_, and 1 mM NaF. After sonication, protein concentrations were determined using a DC protein assay kit (#5000112; Bio-Rad). The proteins were denatured by heating for 10 min at 95 °C, separated on SDS-polyacrylamide gels, transferred onto nitrocellulose membranes (#66485; Pall, New York, NY, USA), and blocked with 5% non-fat milk in Tris-buffered saline with 0.1% Tween 20 (TBST) for 1 h at 25 °C. Next, the blots were incubated with primary antibodies overnight at 4 °C. Unbound antibodies were removed by washing with TBST, and the blots were incubated with the corresponding horseradish peroxidase-conjugated secondary antibodies (#C04001 and #C04003; Croyez Bioscience, Taipei City, Taiwan) for 1 h. Western Lighting ECL detection kits (#1705060; PerkinElmer, Waltham, MA, USA) were used to visualize the protein bands, and the luminance signals were detected using an Amersham Imager 600 (GE Healthcare Life Sciences, Chicago, IL). Images were quantified using ImageJ software (1.52j). The following primary antibodies were used for Western blotting: HIF-1α (GTX127309; GeneTex, Irvine, CA, USA), phospho-YAP (Ser127) (#13008; Cell Signaling Technologies), YAP (GTX60554; GeneTex), phospho-MST1/2 (Thr183/Thr180) (#49332; Cell Signaling Technologies), MST1 (#3682; Cell Signaling Technologies), phospho-LATS1 (Thr1079) (#8654; Cell Signaling Technologies), LATS1 (#3477; Cell Signaling Technologies), phospho-ERK1/2 (Thr202/Tyr204) (#52277; Arigo, Hsinchu City, Taiwan), ERK2 (sc-1647; Santa Cruz, Dallas, TX, USA), phospho-Src (Tyr416) (CST2101; Cell Signaling Technologies), Src (CST2109; Cell Signaling Technologies), phospho-AKT (Ser473) (CST9271; Cell Signaling Technologies), AKT (CST9272; Cell Signaling Technologies), PARP (GTX100573; GeneTex), Caspase 3 (#ab32042; Abcam), α-tubulin (#ab52866; Abcam), and β-actin (A2066; Sigma-Aldrich), lamin B1 (#a16909; Abclonal, Woburn, MA, USA), and GAPDH (#ab8245; Abcam).

### Immunoprecipitation

Extracted protein (1 mg) was immunoprecipitated overnight with HIF-1α (GTX127309; GeneTex) or YAP antibody (#ab52771; Abcam). The following day, the mixture was incubated with protein A/G magnetic beads (LSKMAGAG02; Millipore, Burlington, MA, USA) at 4 °C for 1 h. The supernatant was discarded, and the beads were washed twice with cold PBS containing 0.1% Tween 20 and boiled in sample buffer (10% SDS, 1 M Tris-HCl, 30% glycerol, 6% 2-mercaptoethanol, and 0.12% bromophenol blue in RIPA buffer) for 10 min. The boiled supernatant was transferred to a clean eppendorf tube and subjected to Western blot analysis. The protocol for the negative IgG control groups was the same as the above, except that the antibody used for immunoprecipitated overnight was IgG antibody (#ab172730; Abcam).

### Proximity ligation assay (PLA)

PLA was performed to visualize protein-protein interaction in MDCK cells. In brief, cells were seeded in 3-cm glass-bottom dishes and treated with or without hypoxia-mimetic agent CoCl_2_ for 8 h. After treatment, cells were fixed with 4% paraformaldehyde for 15 min and permeabilized with 0.5% Triton X-100 for 10 min at room temperature. After blocking with CAS-Block (#00-8120; Invitrogen) for 1 h, cells were incubated with primary antibodies (HIF-1α [#GTX127309; GeneTex] and YAP [#H00010413; Abnova]) at 4 °C overnight. On the next day, dishes were washed twice with washing buffer (#DUO82049, Sigma-Aldrich), then incubated with PLA probes (1:5 in antibody diluent) for 1 h at 37 °C. After PLA probes incubation, dishes were then incubated with ligation solution for 30 min at 37 °C, followed by the amplification solution for 100 min at 37 °C. Hoechst 33342 were then added to the dishes for 15 min. The images were acquired using a confocal microscope (FV3000; Olympus).

### 3D cell culture

0.9 mg/ml collagen gel solution (collagen I, #356236; Corning, NY, USA) was prepared on ice. MDCK cells were then mixed with the collagen gel solution in a density of 5 × 10^5^ per 3 cm glass-bottom dish at room temperature and added to the dish. The dishes were then put into the incubator at 37 °C immediately to accelerate gel polymerization. These cell-in-gel dishes were then treated with or without hypoxia-mimetic agent CoCl_2_ for 8 h, followed by the IF staining protocol. The images were captured using a confocal microscope (FV3000; Olympus).

### Statistical analysis

All experiments were repeated at least three times and data were presented as the mean ± SEM. One-way analysis of variance (ANOVA) and two-tailed *t* test were conducted using SPSS software (version 17.0; SPSS Inc., Chicago, IL, USA). The level of significance was set at *p* < 0.05.

### Reporting summary

Further information on research design is available in the Nature Research Reporting Summary linked to this article.

### Supplementary information

Supplementary figures

Original data files

Reporting Summary

## Data Availability

All data generated or analysed during this study are included in this published article and its supplementary information files.
