# Supplementary material for: YAP nuclear translocation induced by HIF-1α prevents DNA damage under hypoxic conditions
Source: Cell Death Discov. 2023 Oct 20;9:385. doi: 10.1038/s41420-023-01687-5 (PMC10589224; doi:10.1038/s41420-023-01687-5)

Supplementary Information

Supplementary Figure 1. $CoCl_2$ induces HIF-1α to interact with YAP, but not TAZ, under high-cell density.

A The quantification of the proportion of YAP localization in Figure 1A was shown. Data are presented as mean ± SEM. $^{**}p < 0.01$ vs. control group under high-cell density conditions. **B** Quantified nuclear fluorescence intensities of HIF-1α and YAP of Figure 1B were depicted. **C** MDCK cells at low density (5×10^4) and high density (3×10^5) were treated with or without 400 μM $CoCl_2$ for 8 h. Immunofluorescent images of TAZ (red) and HIF-1α (green) were taken using confocal microscope. Scale bar: 10 μm.

Supplementary Figure 2. Hypoxia also promotes HIF-1α and YAP interaction in HK-2 cells.

A–B HK-2 cells at high density (3×10^5) were treated with or without 400 μM $CoCl_2$ for 8 h. Immunofluorescent images of YAP (red) and HIF-1α (green) were taken using confocal microscope. Scale bar: 10 μm. The proportion of YAP localization in cytoplasmic, nuclear, or both cytoplasmic and nuclear at low and high density were quantified in (**B**). **C** HK-2 cells at low and high density were treated with or without 1% O_2 for 8 h. Protein levels of Hippo pathway in each group were determined by

Western blotting. The ratio of phospho-MST1/MST1, phospho-LATS1/LATS1, and phospho-YAP/YAP were listed below the corresponding blots. **D** High-density HK-2 cells were transient transfected with shHIF-1α, and subjected to 1% O_2 for 24 h, followed by immunostaining with anti-γH2AX antibody. Scale bar: 10 μm. **E** High-density HK-2 cells were transient transfected with shHIF-1α, and subjected to 1% O_2 for 36 h, followed by Annexin V staining. Scale bar: 10 μm. All immunofluorescence images were obtained using confocal microscope.

Supplementary Figure 3. Immunoprecipitations showed an increase of HIF-1α and YAP under $CoCl_2$-treated high-density condition.

MDCK cells at low and high density were incubated with or without 400 μM $CoCl_2$ for 8 h. The immunoprecipitations were performed and analyzed by Western blotting with anti-HIF-1α antibody.

Supplementary Figure 4. HIF-1α facilitates YAP downstream gene expression under hypoxic condition.

MDCK-Parental and MDCK-shHIF-1α cells at high density were subjected to 1% O_2 for 0, 2, 4, and 6 h. Representative PCR results of gene expression for *yap* and the downstream gene *cyr61* were shown. *gapdh* was used as an internal control.

Supplementary Figure 5. Effects of Src and ERK inhibitors on YAP localization under $CoCl_2$ treatment.

MDCK cells at high cell density were treated with 1 μM dasatinib, 100 μM U0126, or 1 μM SCH772984 for 24 h, followed by 400 μM $CoCl_2$ for 8 h. **A** Representative Western blots were shown. Quantification of (**B**) phospho-Src/Src, (**C**) phospho-ERK2/ERK2, and (**D**) phospho-YAP/YAP is presented as mean $\pm$ SEM. **B** $**p < 0.01$ vs. the $CoCl_2$-alone group. **C** $*p < 0.05$ vs. control group. $\#\#\#p < 0.001$ vs. control group. $\#\#p < 0.01$ vs. $CoCl_2$-alone group. **D** $**p < 0.01$ and $*p < 0.05$. **E** Immunofluorescence images of YAP (red) and HIF-1α (green) were obtained using confocal microscope. Scale bar: 10 μm. **F** Proportion of YAP localization in cytoplasm and nucleus was quantified. Data are presented as mean $\pm$ SEM. $**p < 0.01$.

Supplementary Figure 6. AKT inhibitor decreases YAP phosphorylation at serine 127 in MDCK-shHIF-1α cells under high-cell density conditions.

High density MDCK-shHIF-1α cells were treated with or without 10 μM LY294002 for 24 h, followed by control or 400 μM $CoCl_2$ treatment for 8 h. **A–B** Immunofluorescence images of YAP (red) and HIF-1α (green) obtained using confocal microscope. Scale bar: 10 μm. Proportion of YAP localization in cytoplasm, nucleus, or both after LY294002 treatment was quantified in (**B**). Bars represent the mean $\pm$ SEM. ***p < 0.001, ###p <

0.001. **C** Representative blots of HIF-1α, phospho-AKT, AKT, phospho-YAP, and YAP were shown. α-tubulin was used as a loading control.

Supplementary Figure 7. DNA distribution of parental and shHIF-1α cells under normoxic and hypoxic conditions.

MDCK-parental and MDCK-shHIF-1α cells were treated with normoxia or 1% O_2 for 48 h and stained with Hoechst 33342. Nuclei morphology was observed under an inverted fluorescence microscope. Pseudocolor shows distribution and intensity of DNA by Hoechst 33342 staining in nucleus. Scale bar: 10 μm.

Supplementary Figure 8. Hypoxic memetic $CoCl_2$ induced DNA damage in MDCK cells.

A–B MDCK-parental and MDCK-shHIF-1α cells were treated with normoxia or 400 μM $CoCl_2$ for 24 h, followed by immunostaining with anti-γH2AX antibody. **A** Representative confocal images were shown. Scale bar: 40 μm. **B** γH2AX intensity was analyzed and presented as mean ± SEM. $***p < 0.001$.

Supplementary Figure 9. Constitutively active YAP reduced DNA damage under hypoxic conditions.

A–B MDCK-shHIF-1α-YAP-WT and MDCK-shHIF-1α-YAP-S127A cells were exposed to 1% O_2 for 24 h, followed by immunostaining with anti-γH2AX antibody. **A** Representative confocal images were shown. Scale bar: 40 μm. **B** γH2AX intensity was analyzed and presented as mean ± SEM. *$p < 0.05$.

Supplementary Figure 10. MDCK cells with either HIF-1α or YAP deficiency were sensitive to hypoxic insults.

A High-density MDCK-Parental, MDCK-shHIF-1α, and MDCK-shYAP cells were subjected to 1% O_2 for 24 h, followed by immunostaining with anti-Rad51 antibody. Scale bar: 10 μm. **B** MDCK-Parental, MDCK-shHIF-1α, and MDCK-shYAP cells were seeded in high density and were treated with 1% O_2 for 36 h. The expression of PARP and caspase 3 were determined by Western blotting. α-tubulin was used as a loading control.

Supplementary Figure 1

A

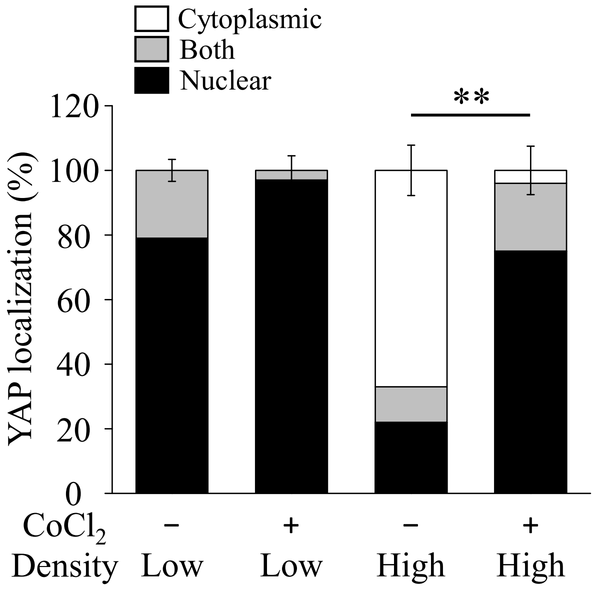

C

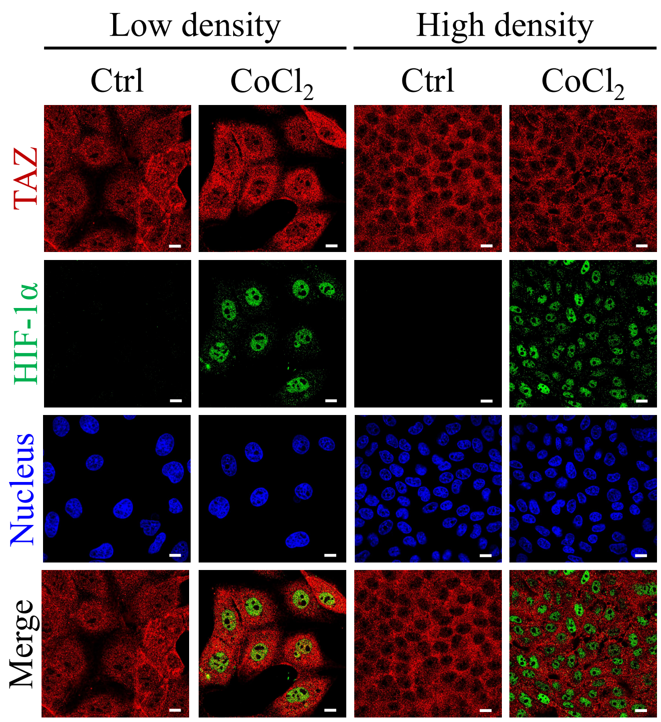

B

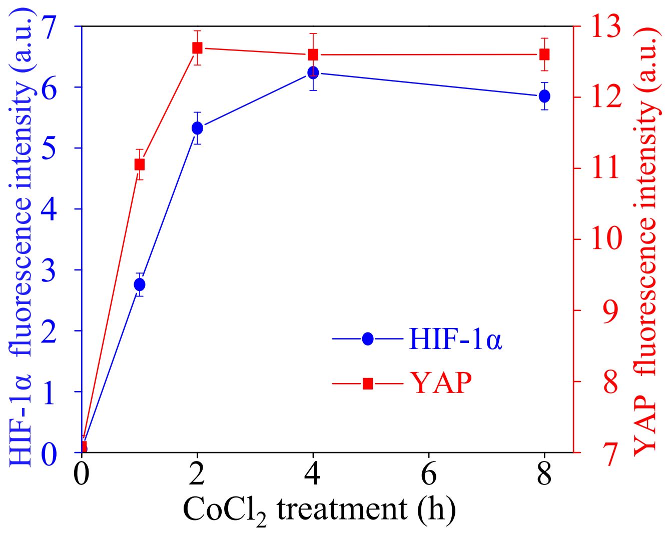

Supplementary Figure 2

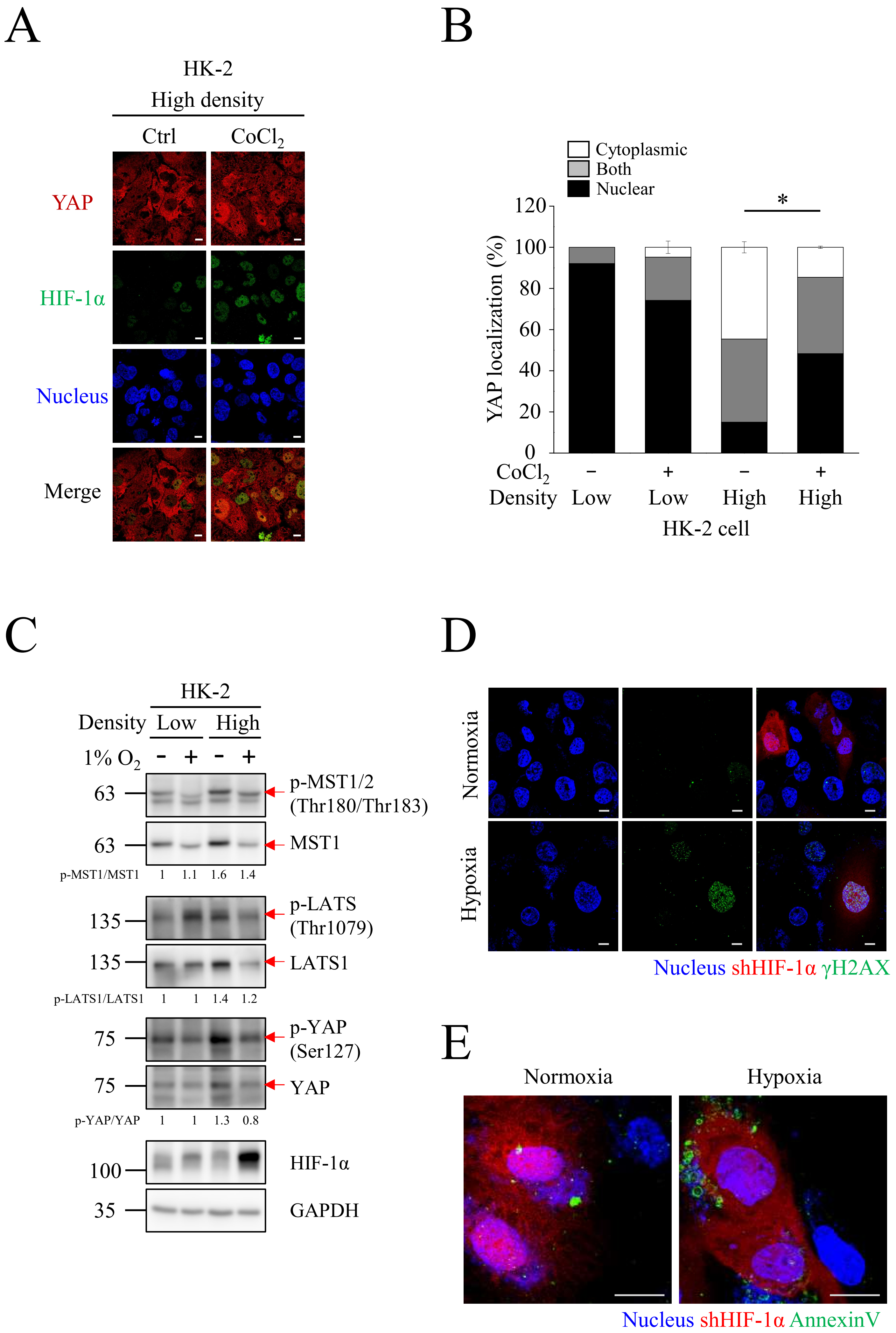

Supplementary Figure 3

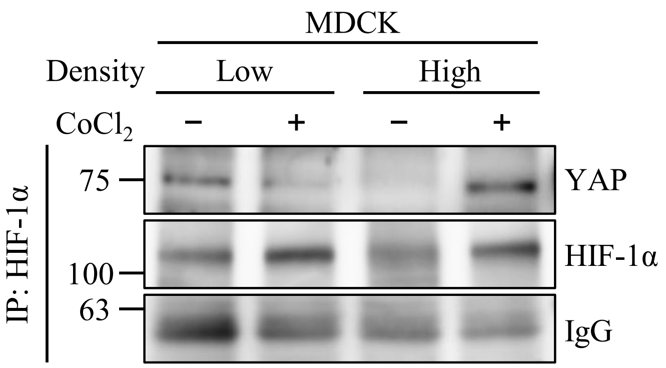

Supplementary Figure 4

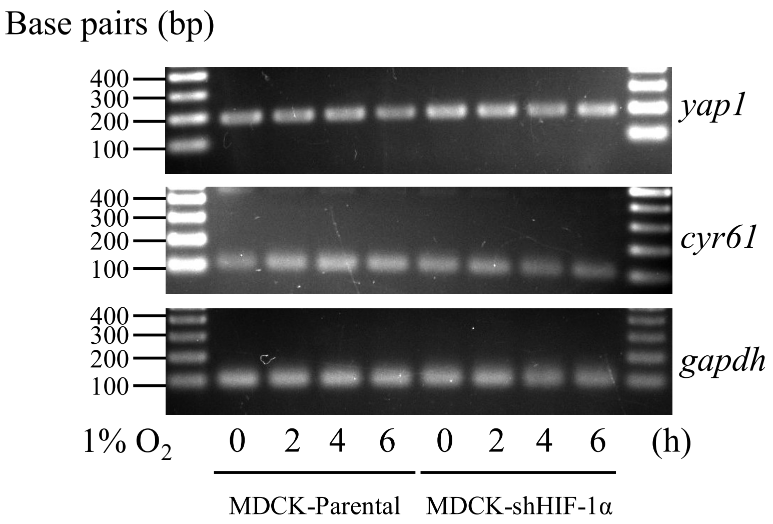

Supplementary Figure 5

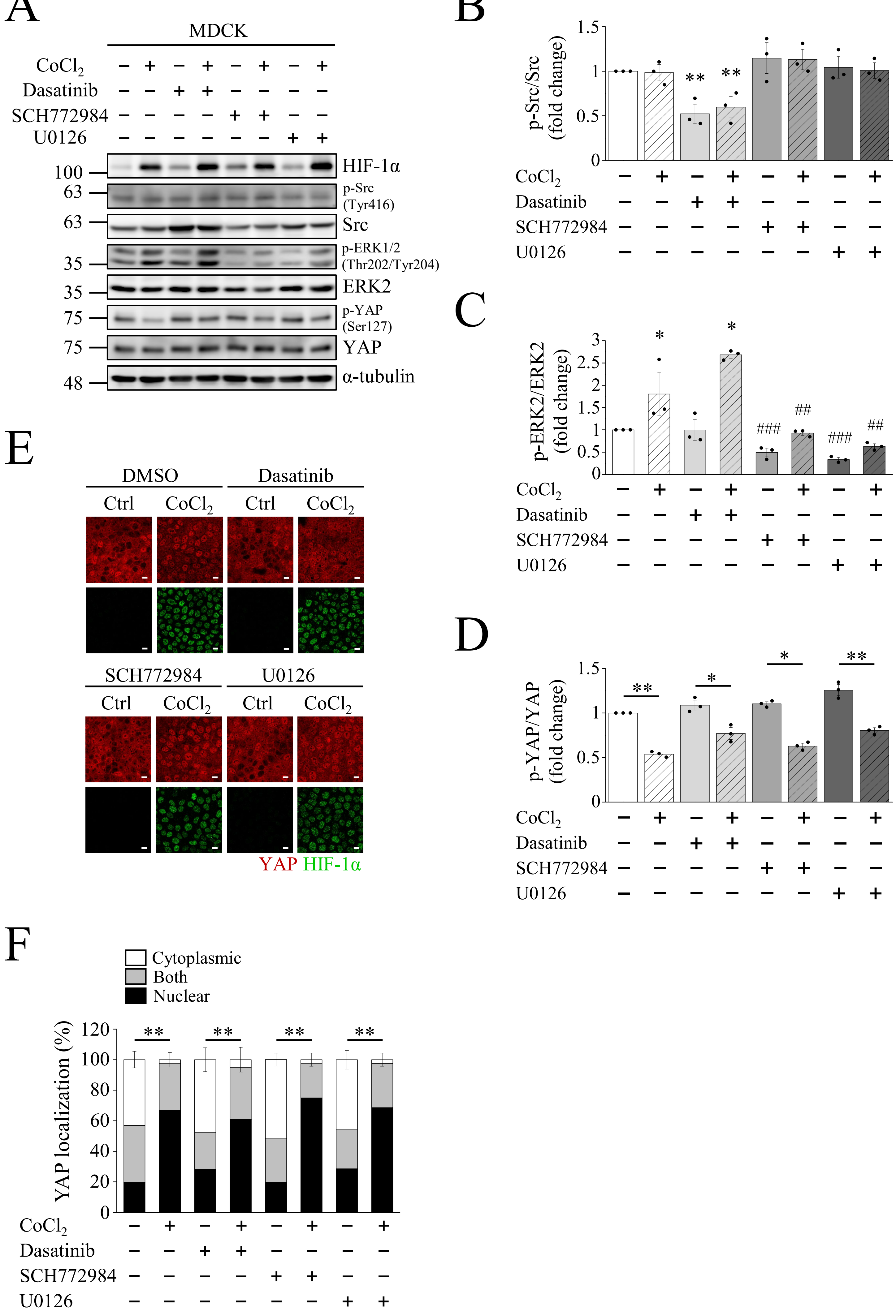

Supplementary Figure 6

A

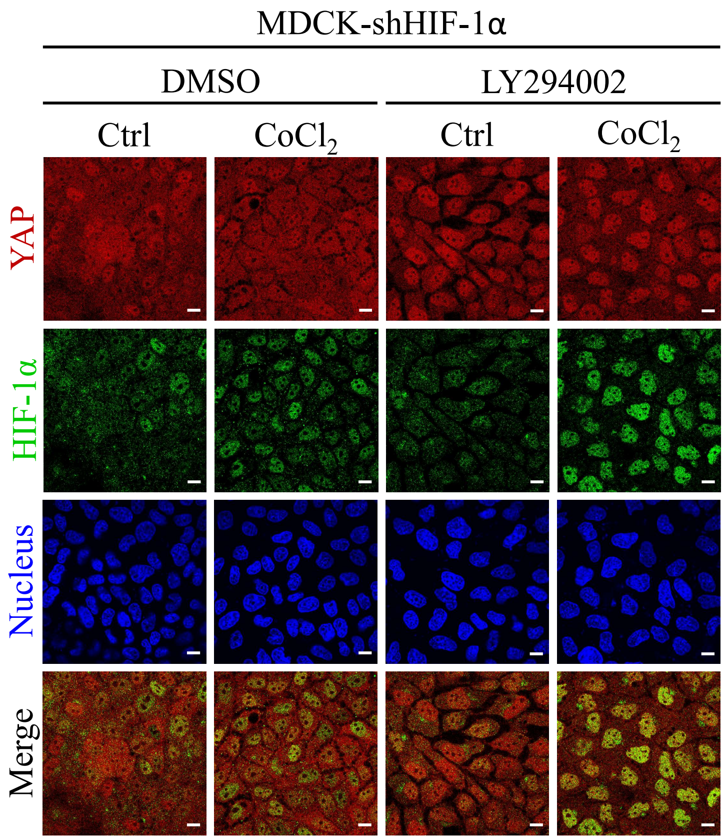

B

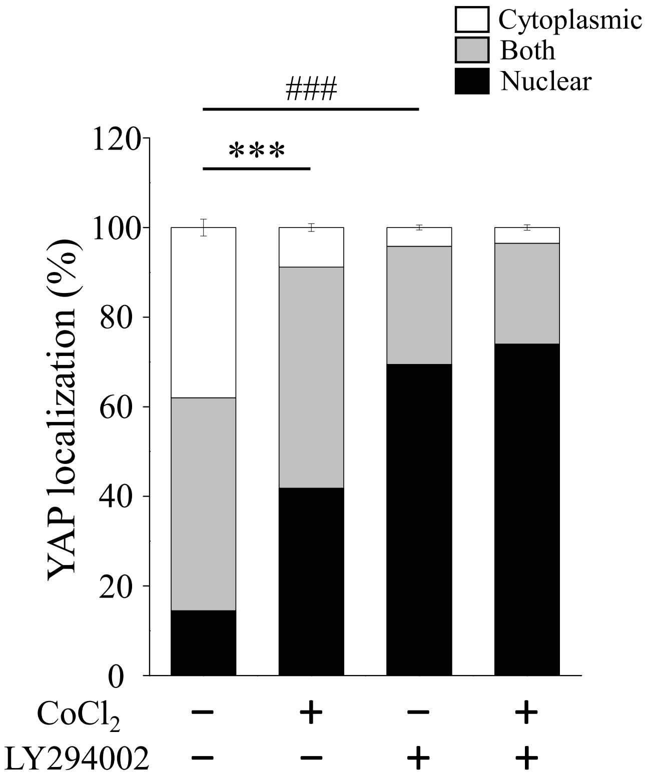

C

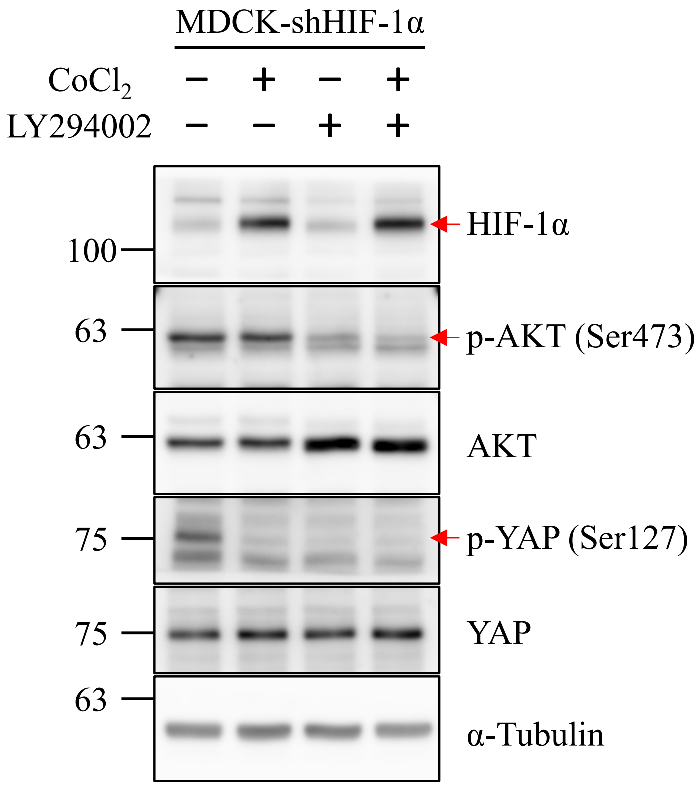

Supplementary Figure 7

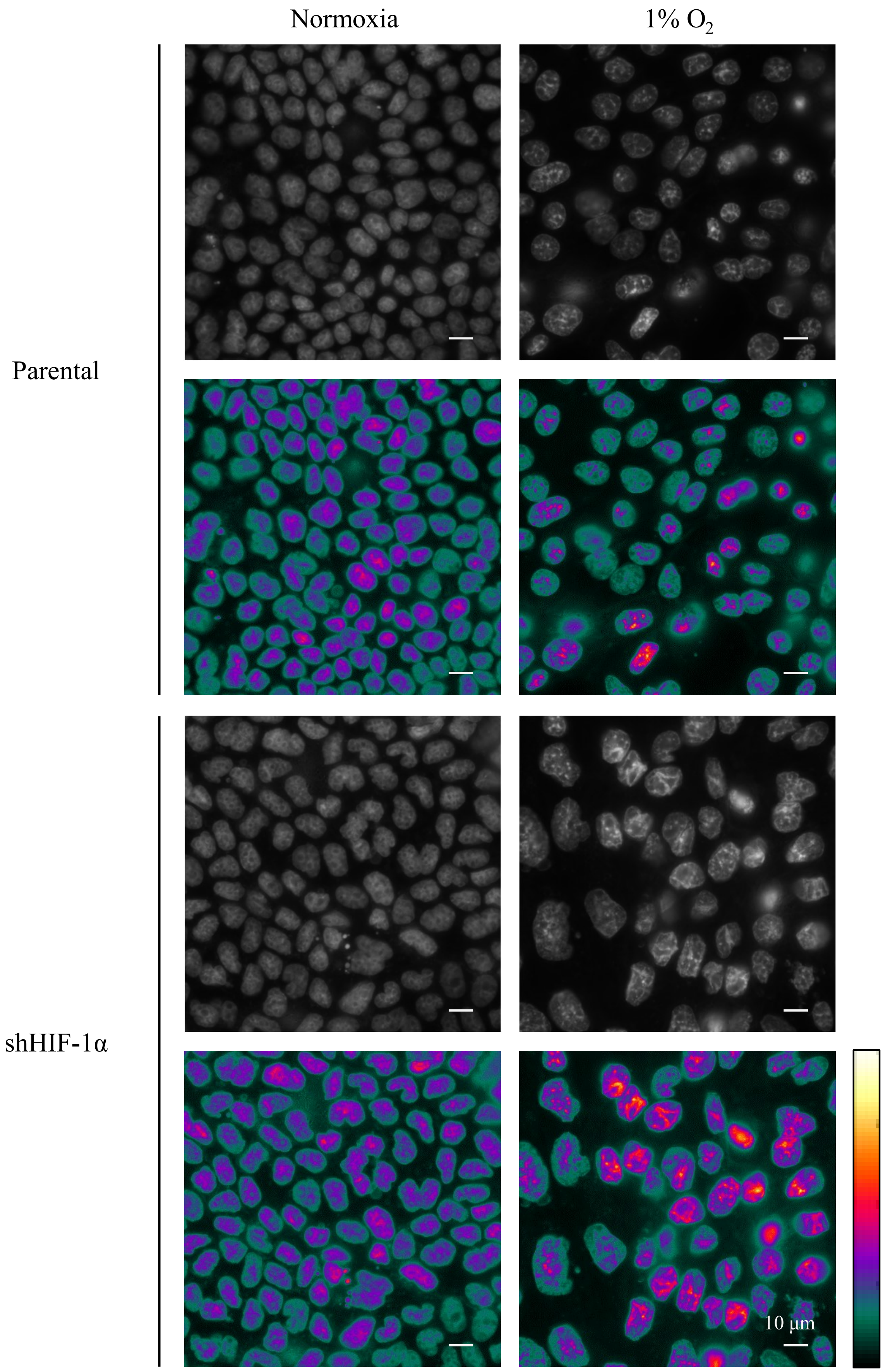

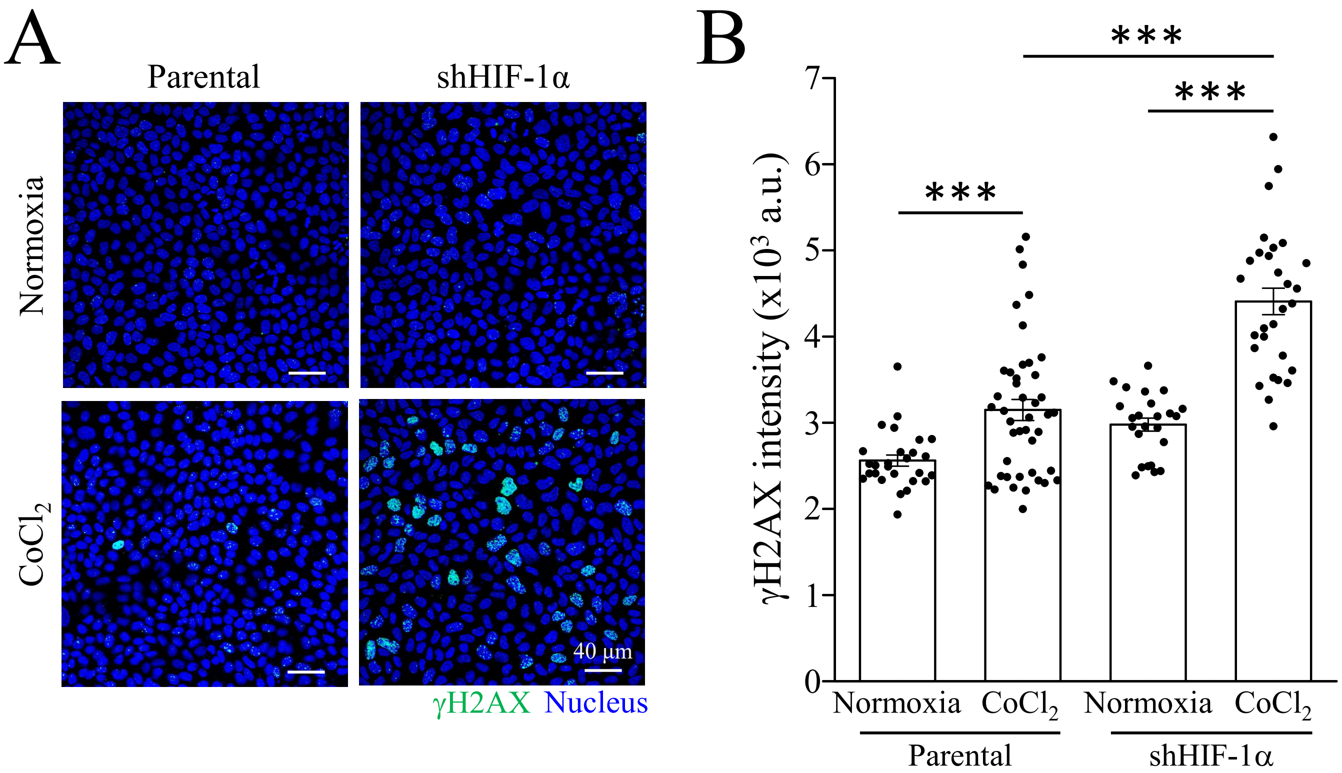
A
Parental
shHIF-1α
Normoxia
CoCl2
40 µm
γH2AX Nucleus
B
γH2AX intensity (x10³ a.u.)
***
***
***
0
1
2
3
4
5
6
7
Normoxia
CoCl2
Normoxia
CoCl2
Parental
shHIF-1α

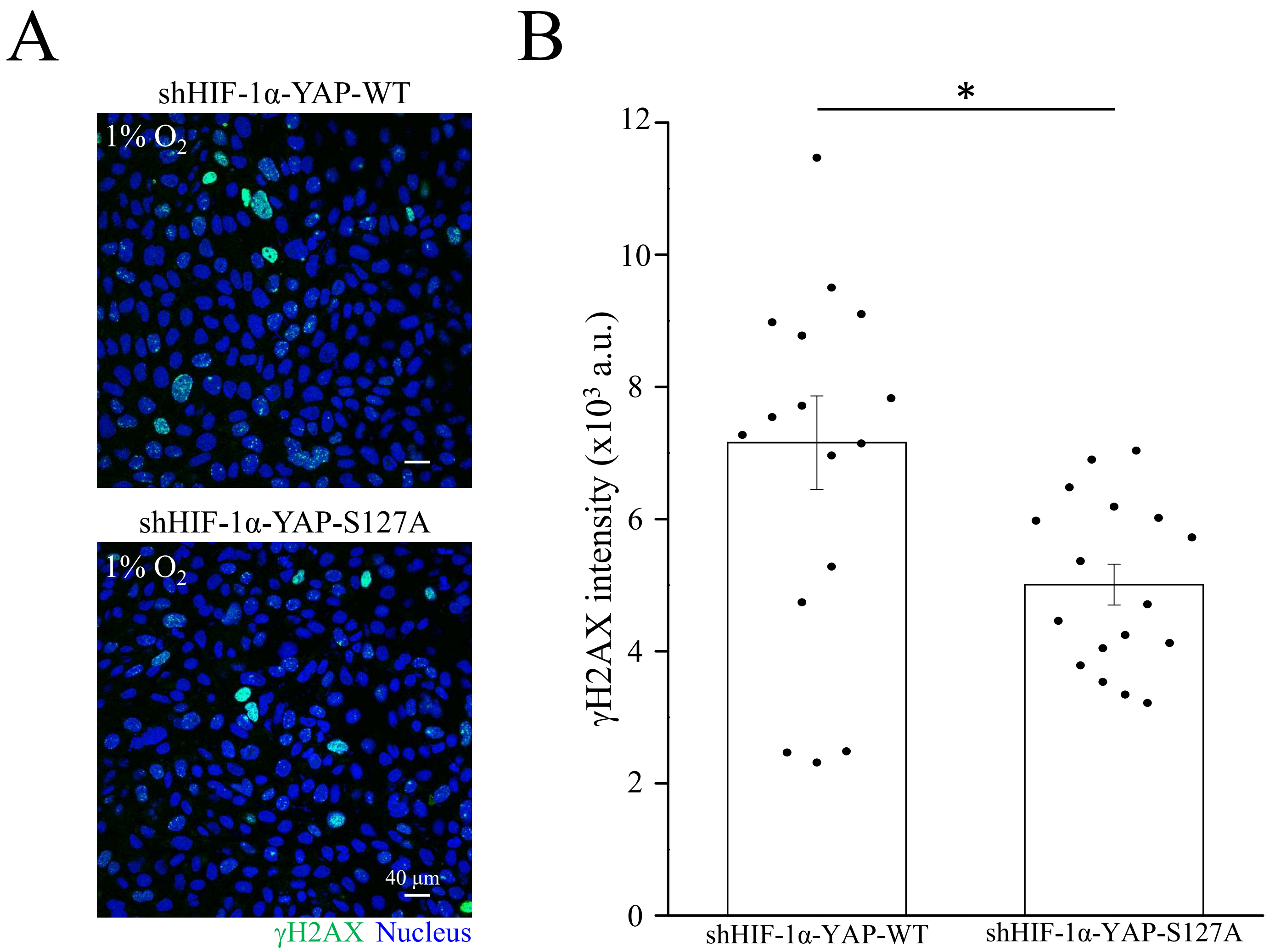

A
shHIF-1α-YAP-WT
1% O2
shHIF-1α-YAP-S127A
1% O2
40 μm
γH2AX Nucleus
B
*
γH2AX intensity (x10³ a.u.)
12
10
8
6
4
2
0
shHIF-1α-YAP-WT
shHIF-1α-YAP-S127A

Supplementary Figure 10

A

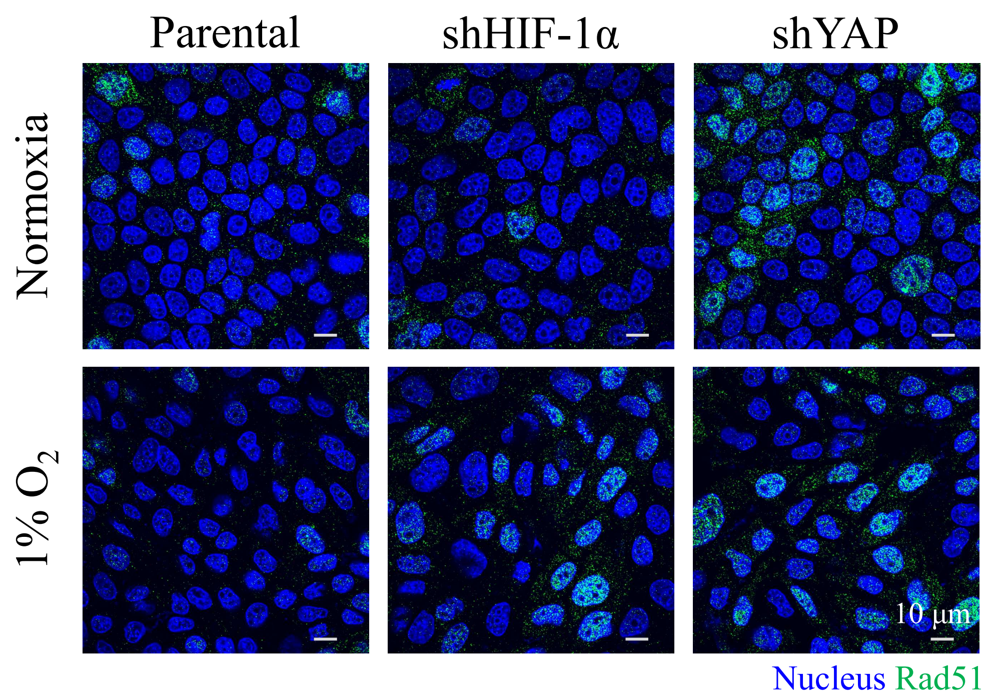

B

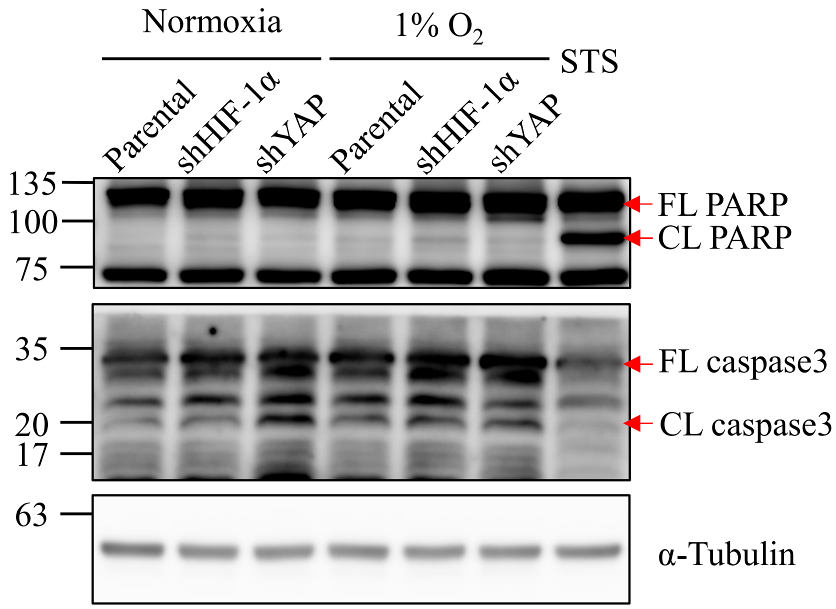

Supplement: Supplementary file 1 — Supplementary figures [file 41420_2023_1687_MOESM1_ESM.pdf]
