# Supplementary figures and images for: YAP nuclear translocation induced by HIF-1α prevents DNA damage under hypoxic conditions
Source: Cell Death Discov. 2023 Oct 20;9:385. doi: 10.1038/s41420-023-01687-5 (PMC10589224; doi:10.1038/s41420-023-01687-5)

Fig 2A

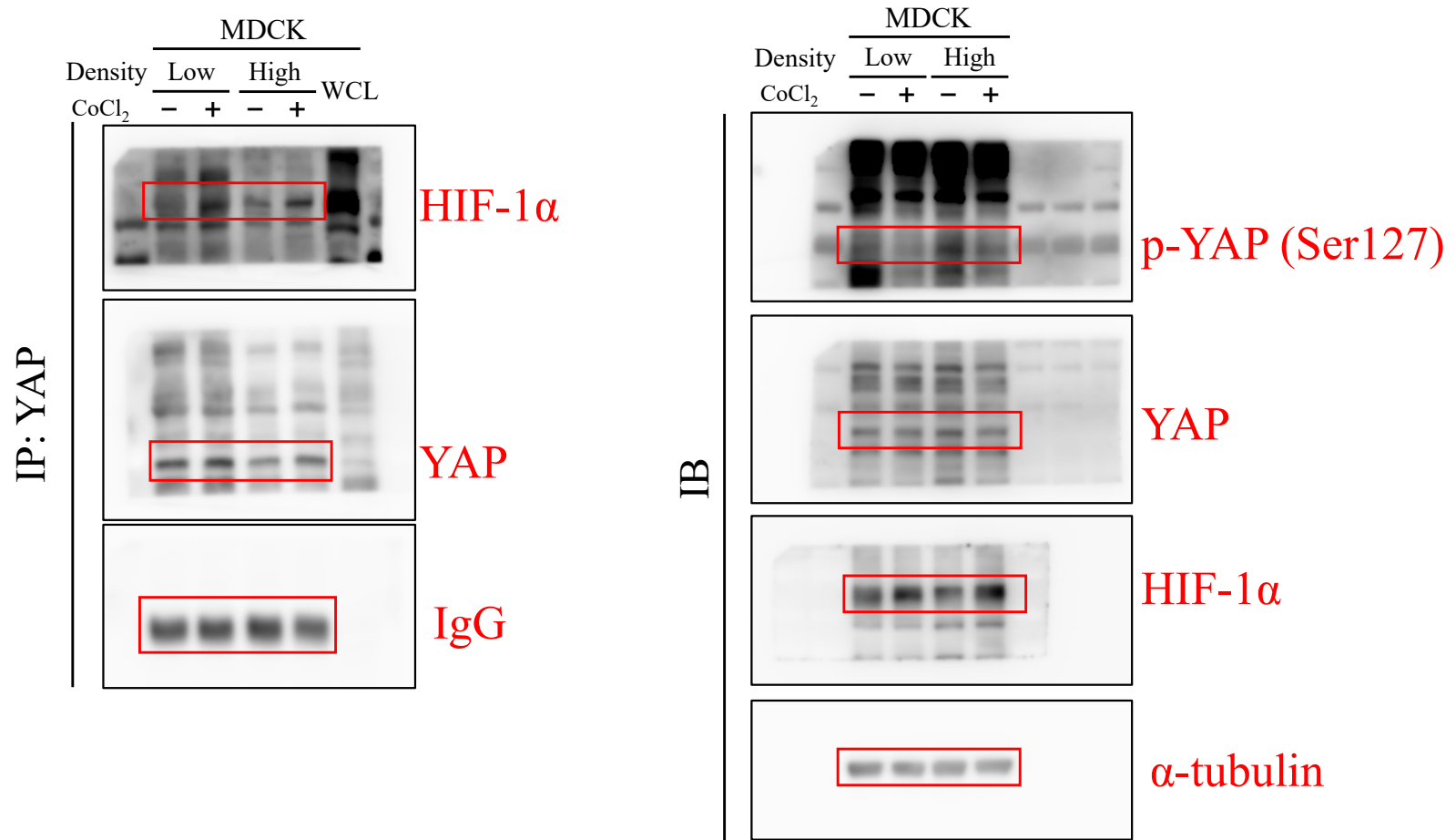

Fig 2B

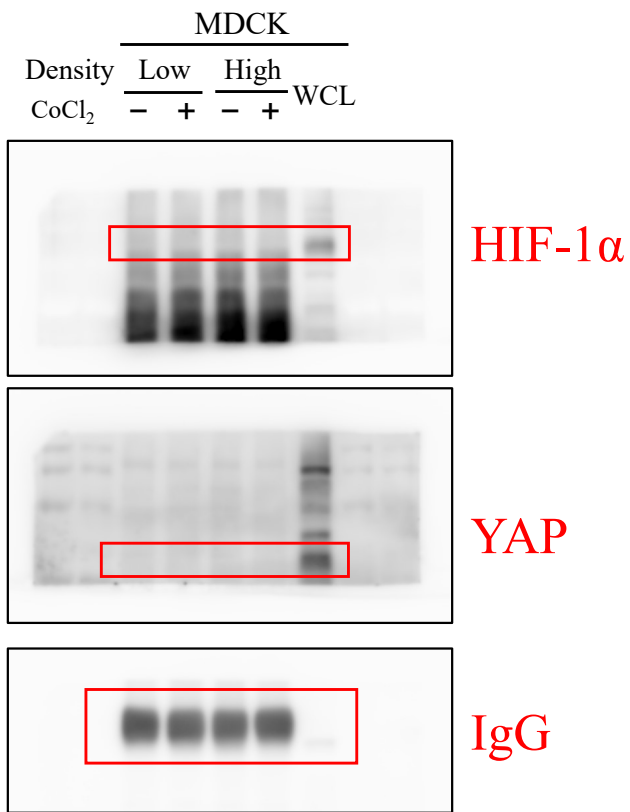

Fig 2C

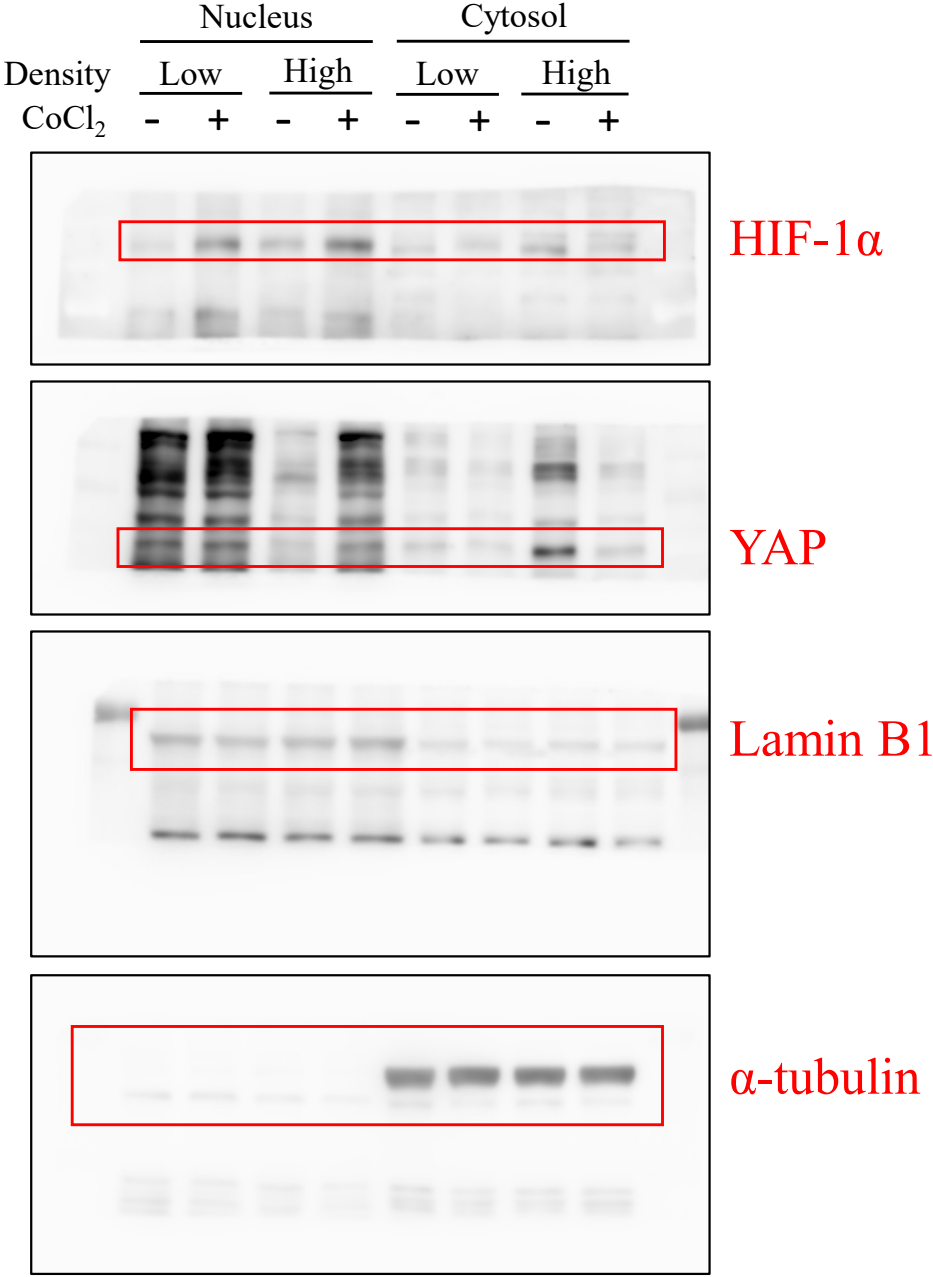

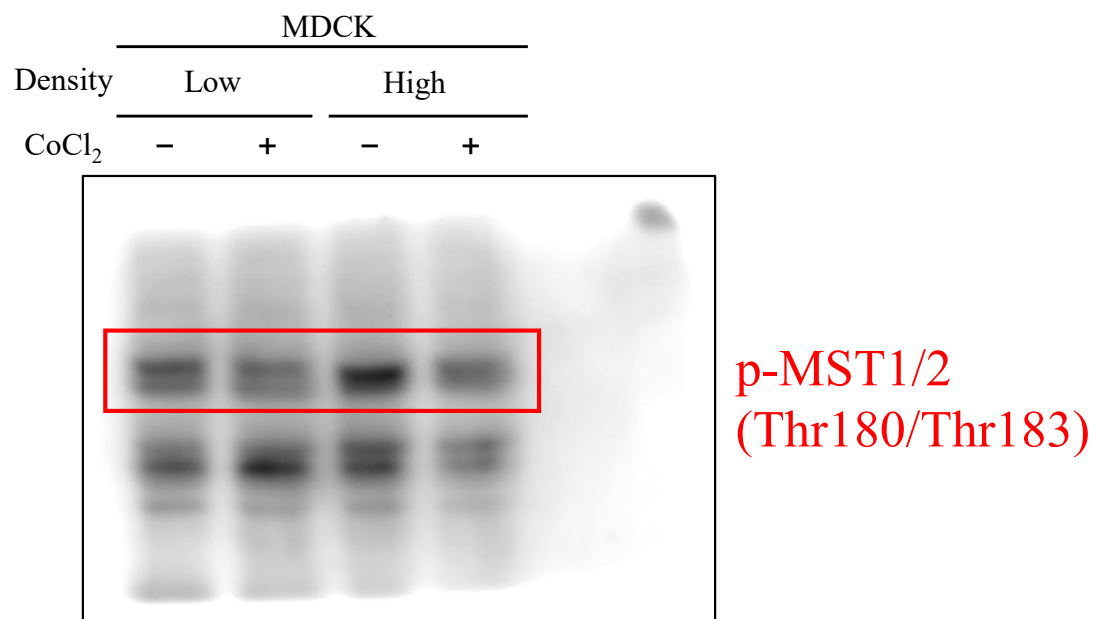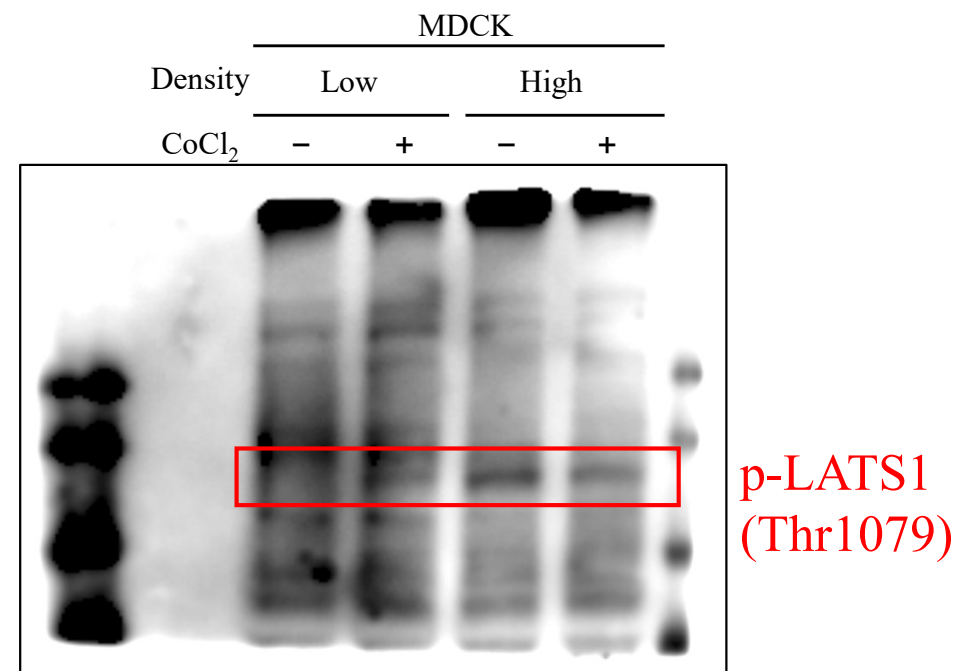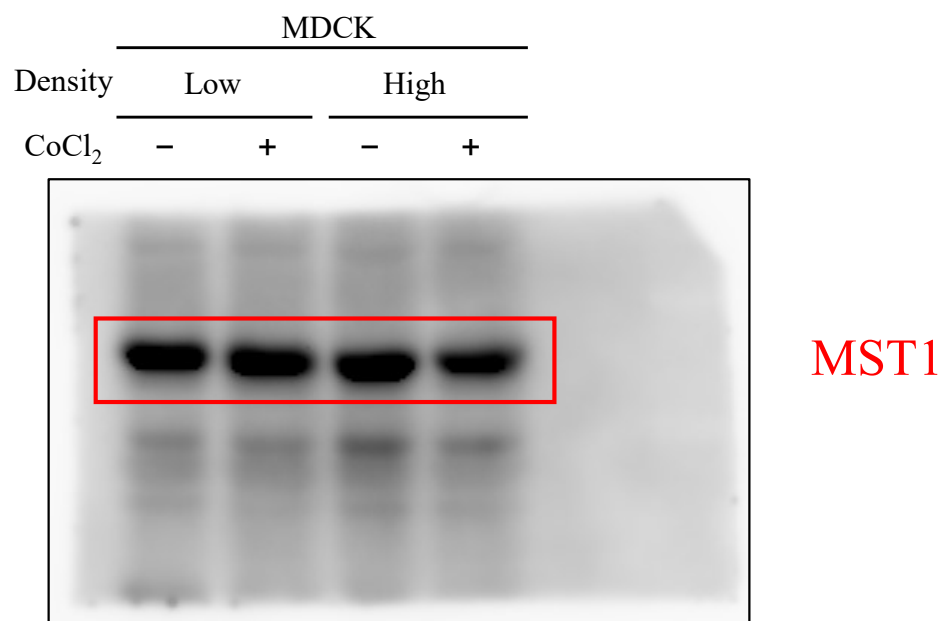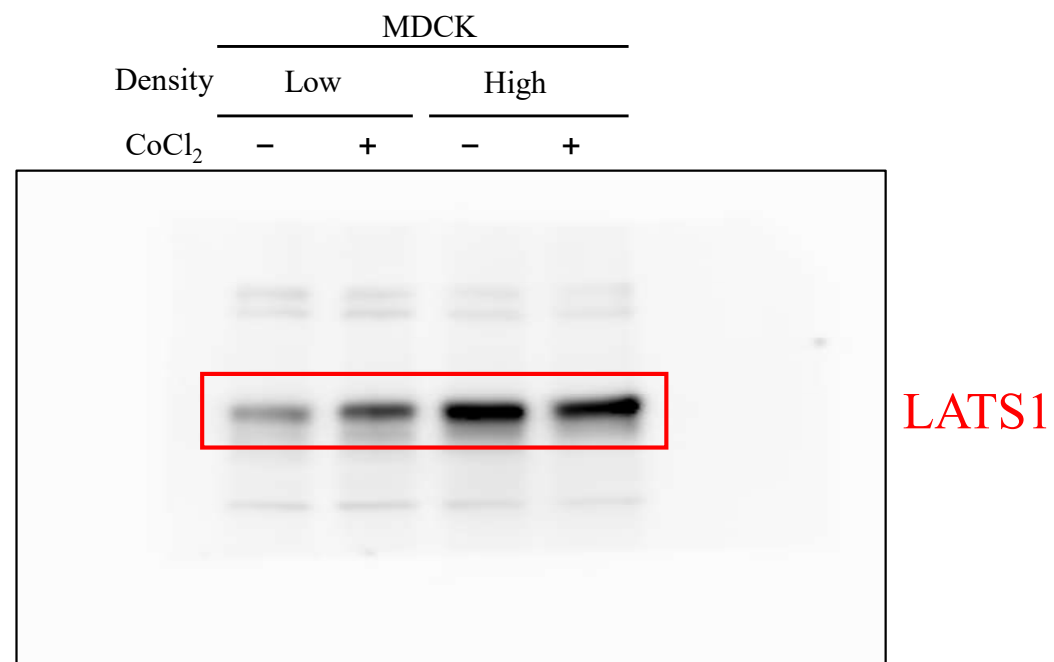

Fig. 3A

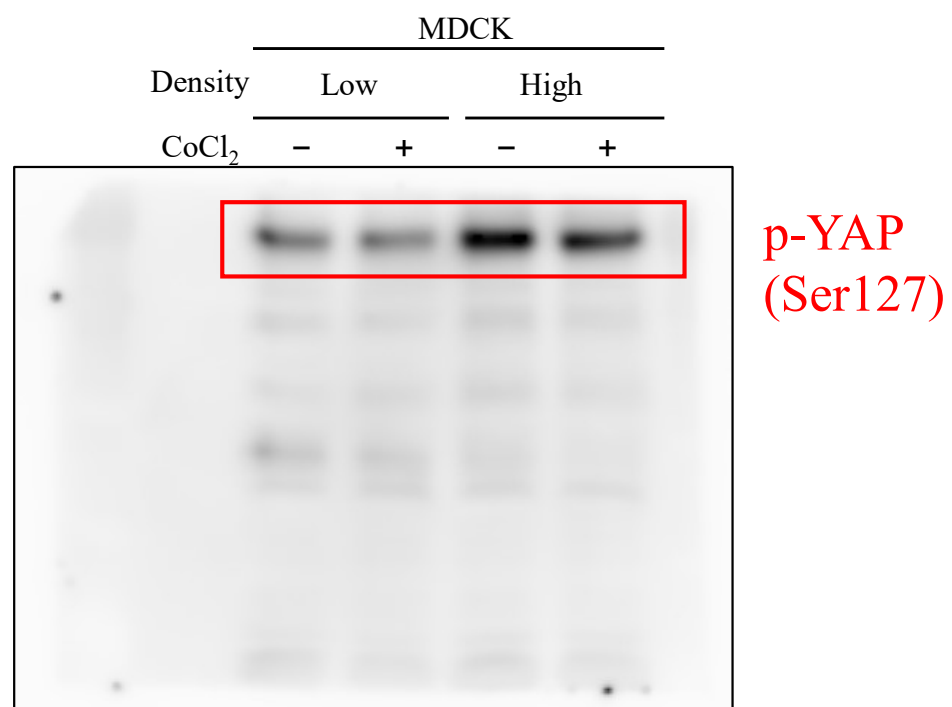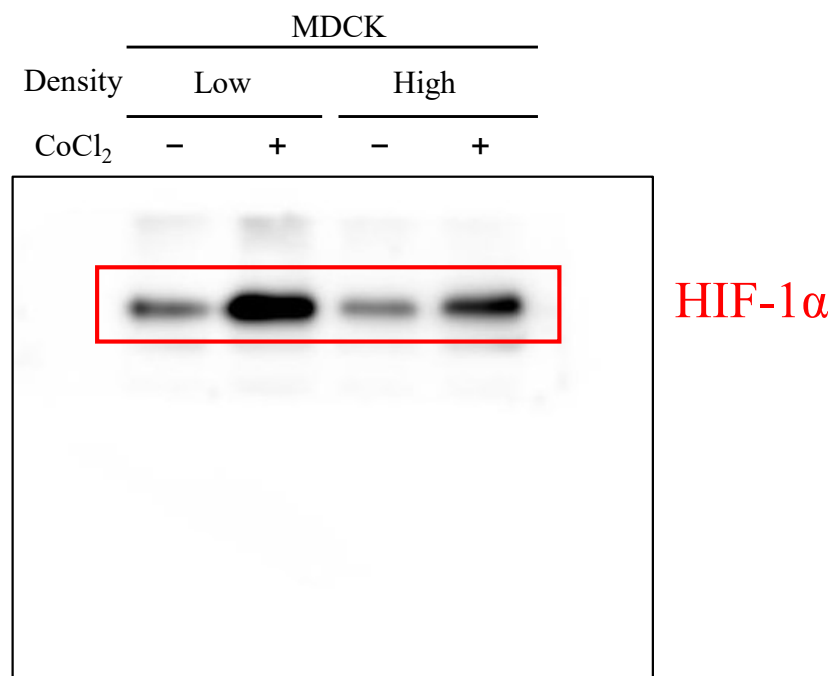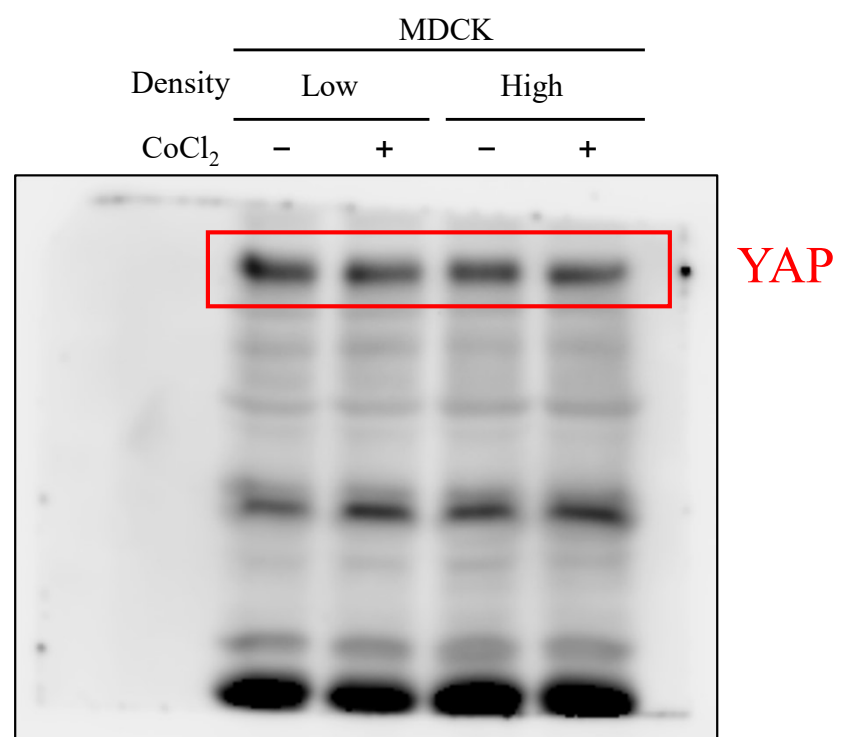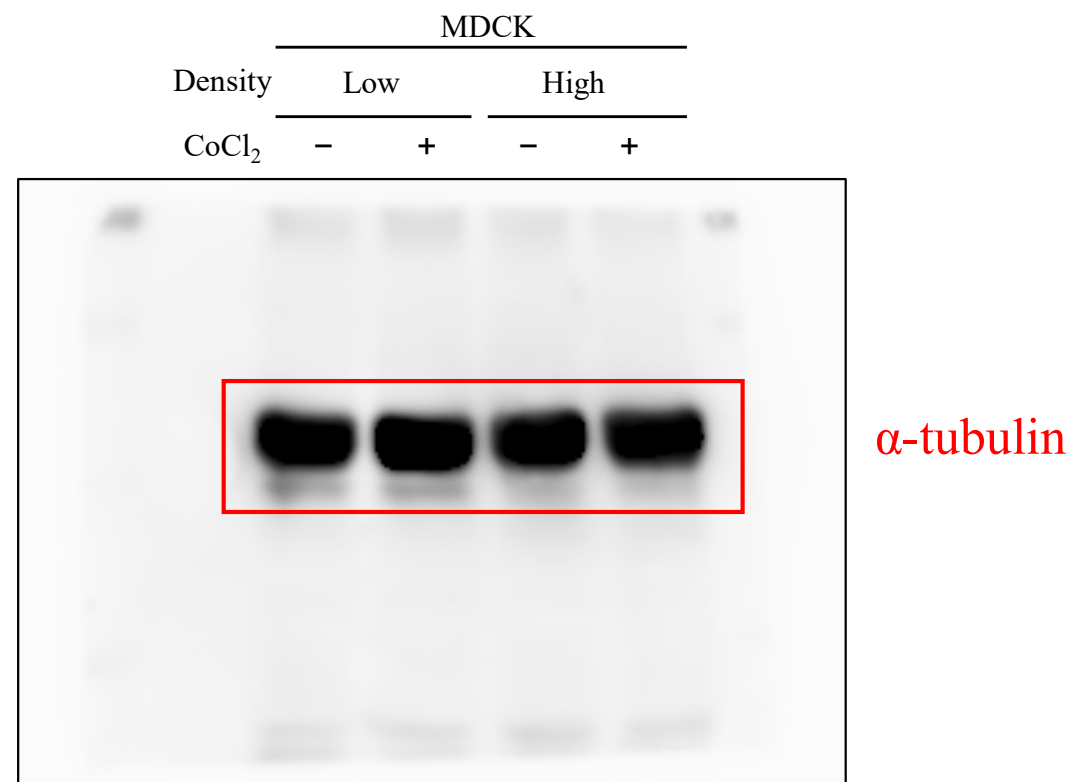

Fig. 4A

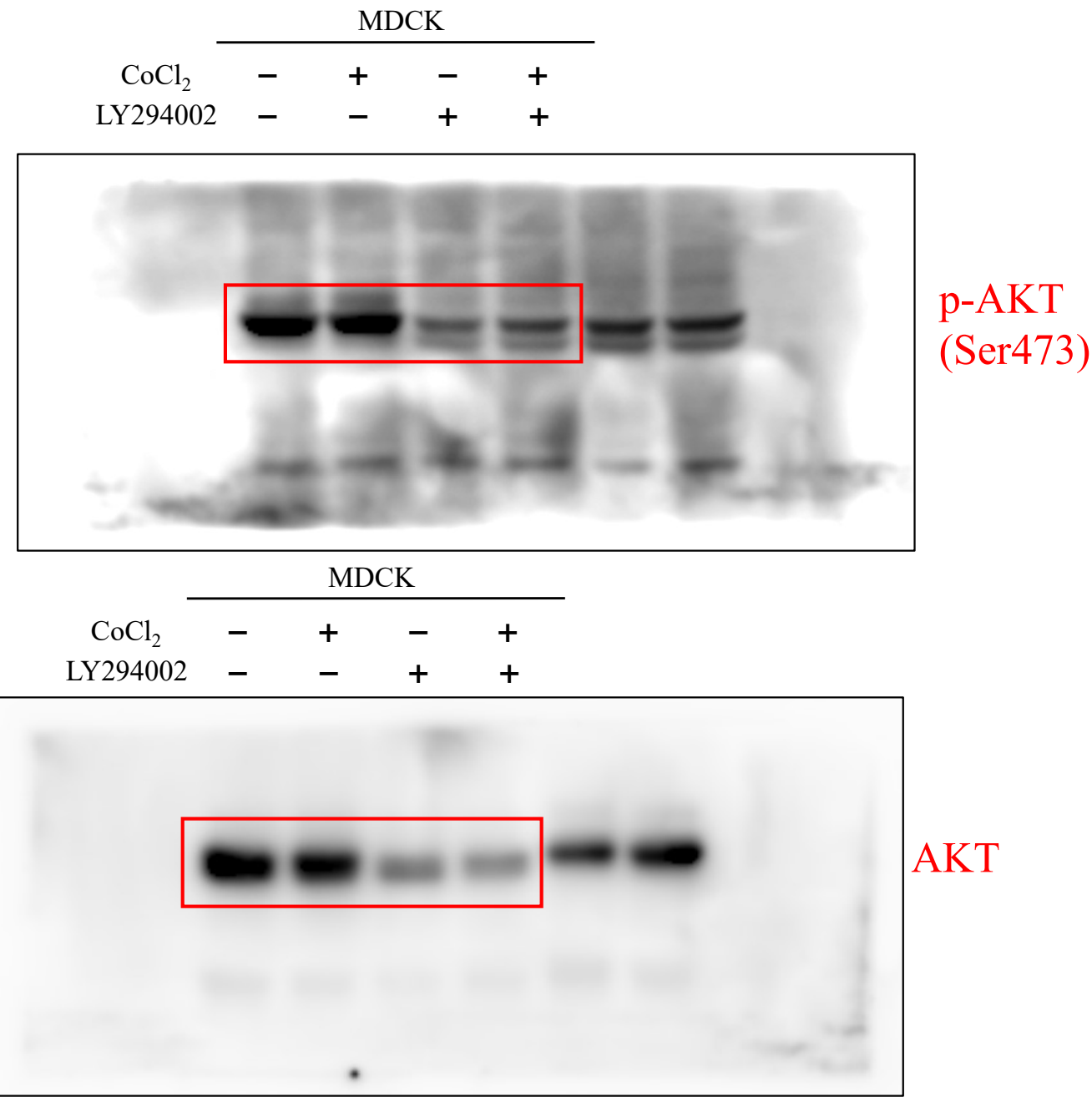

Fig. 4A

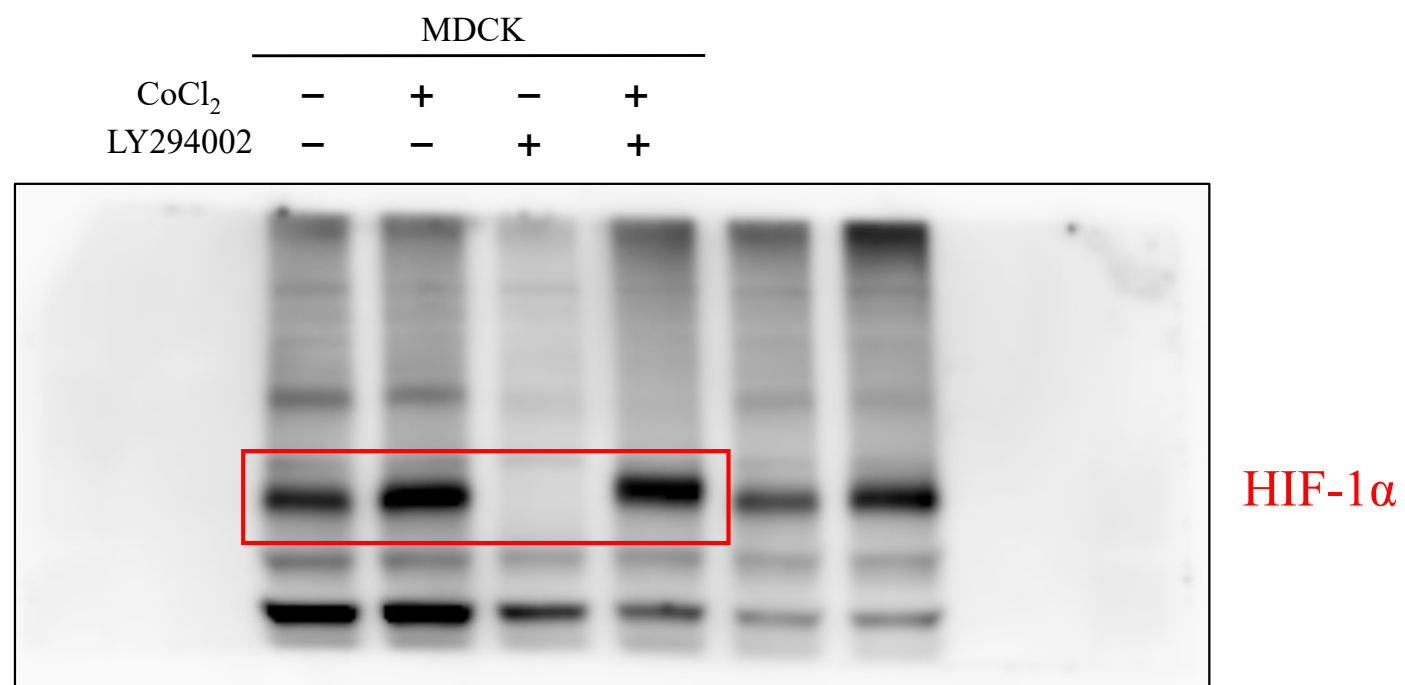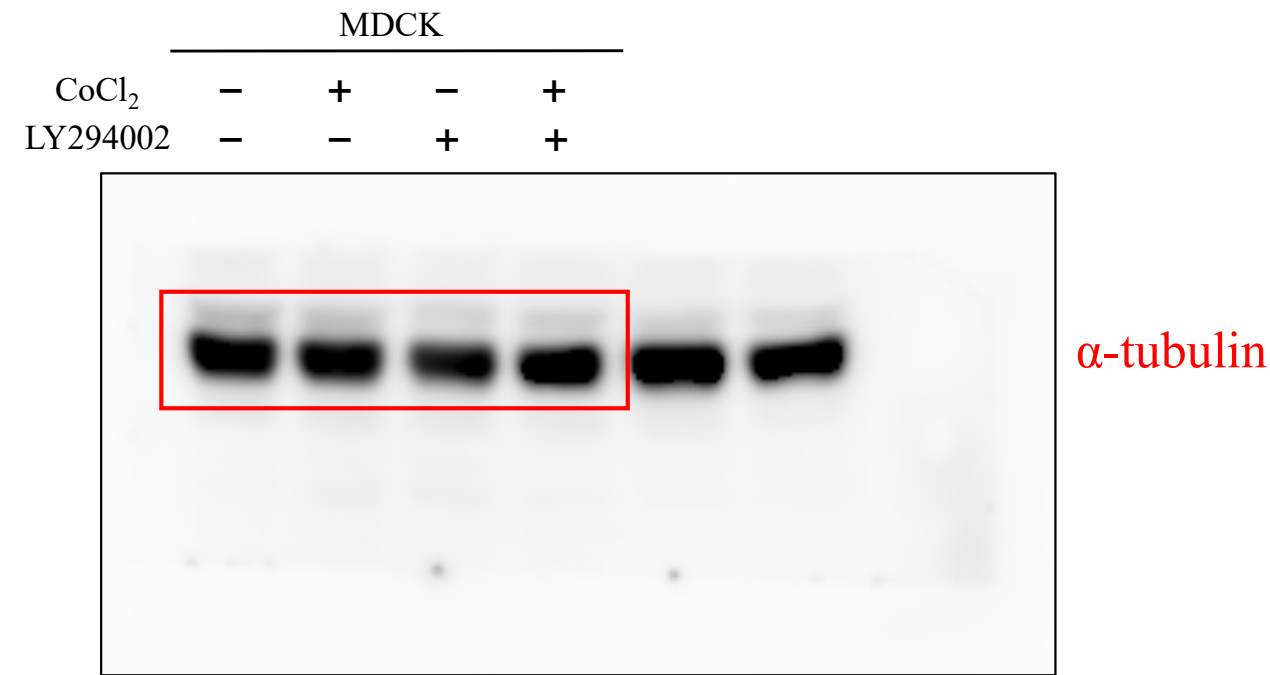

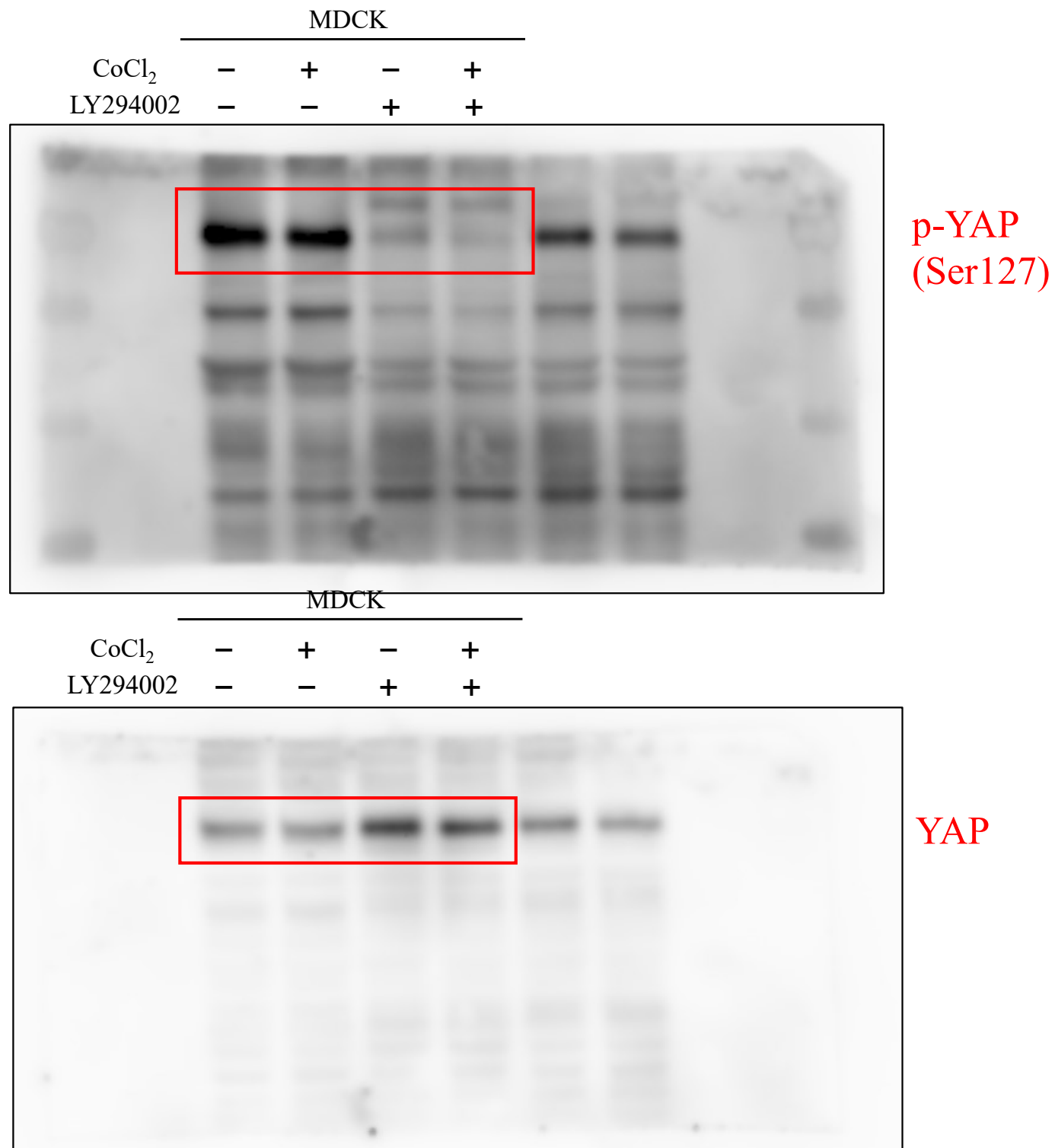

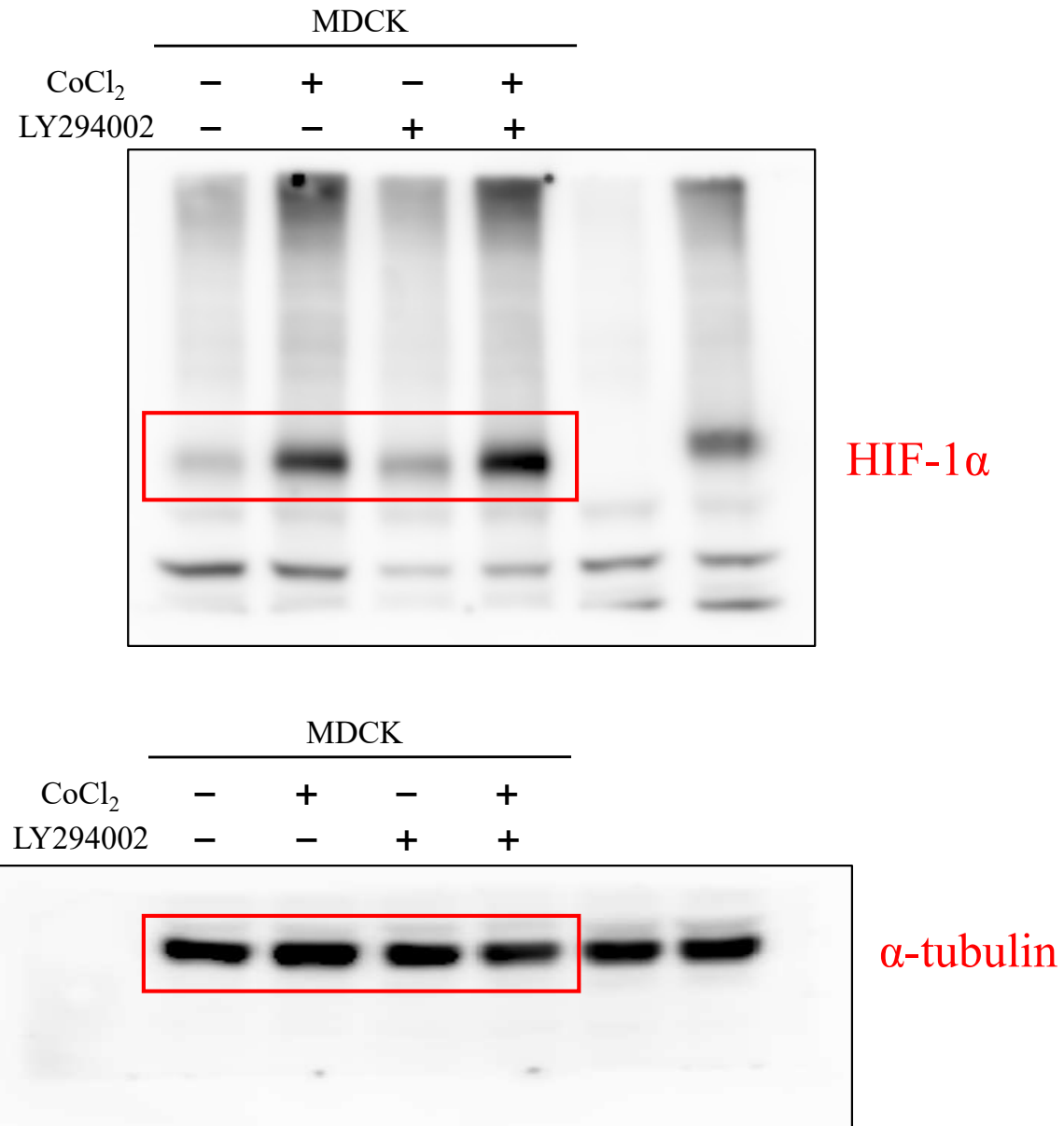

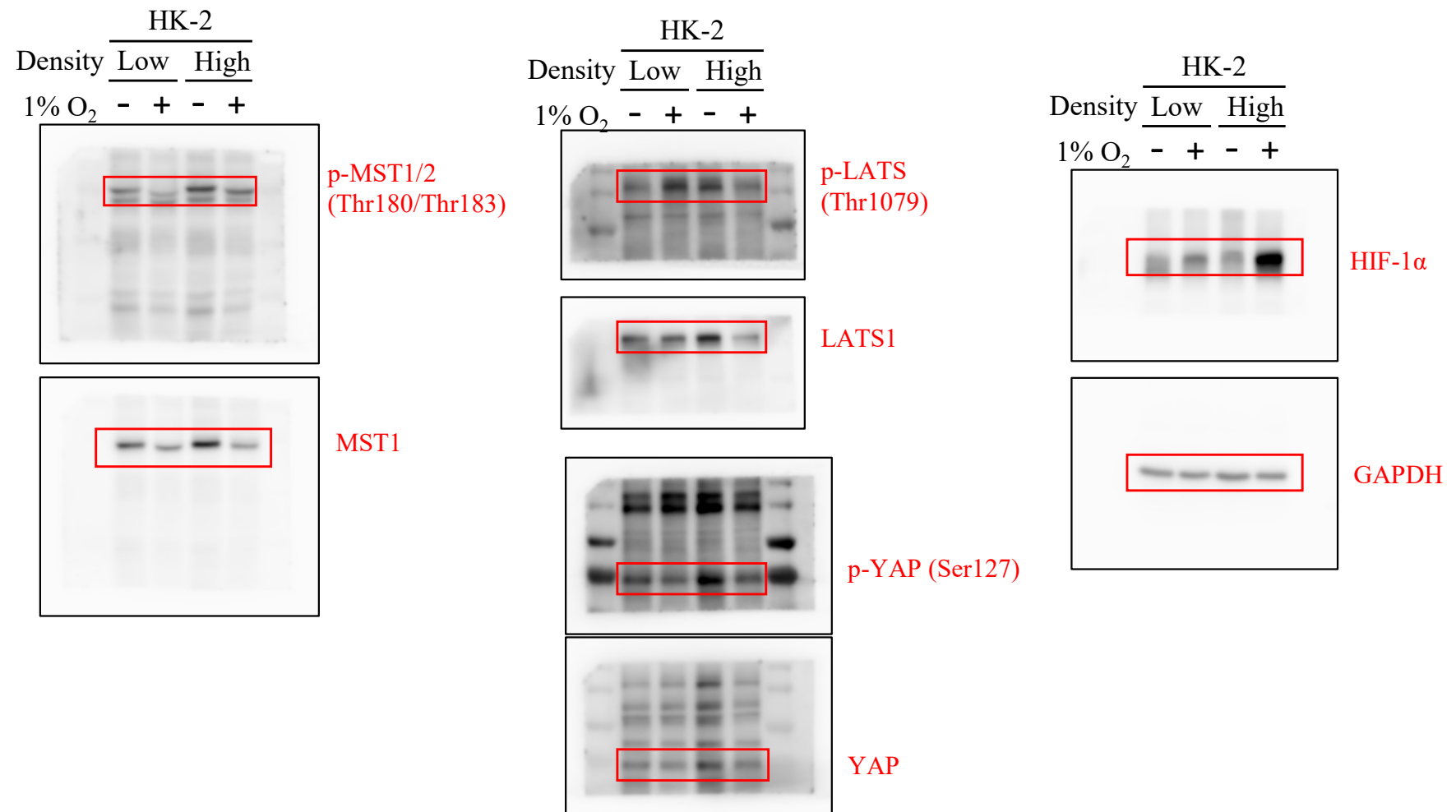

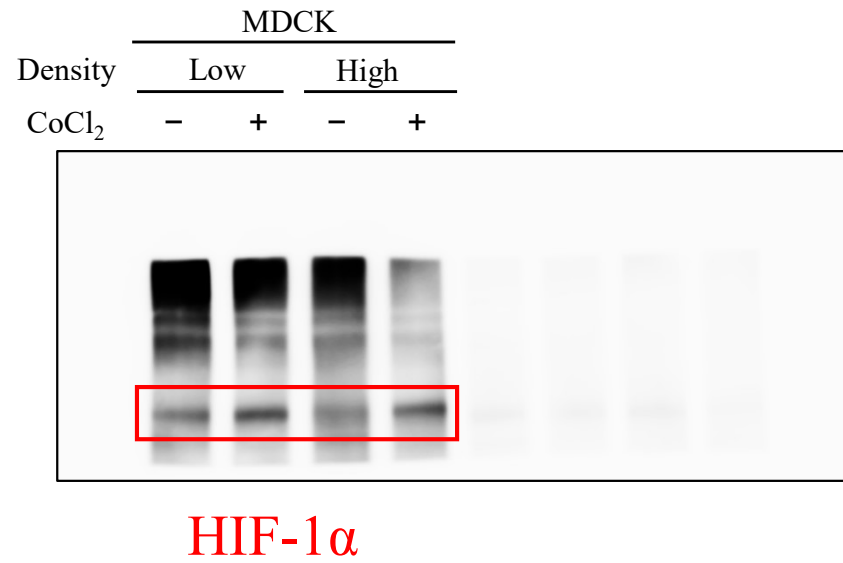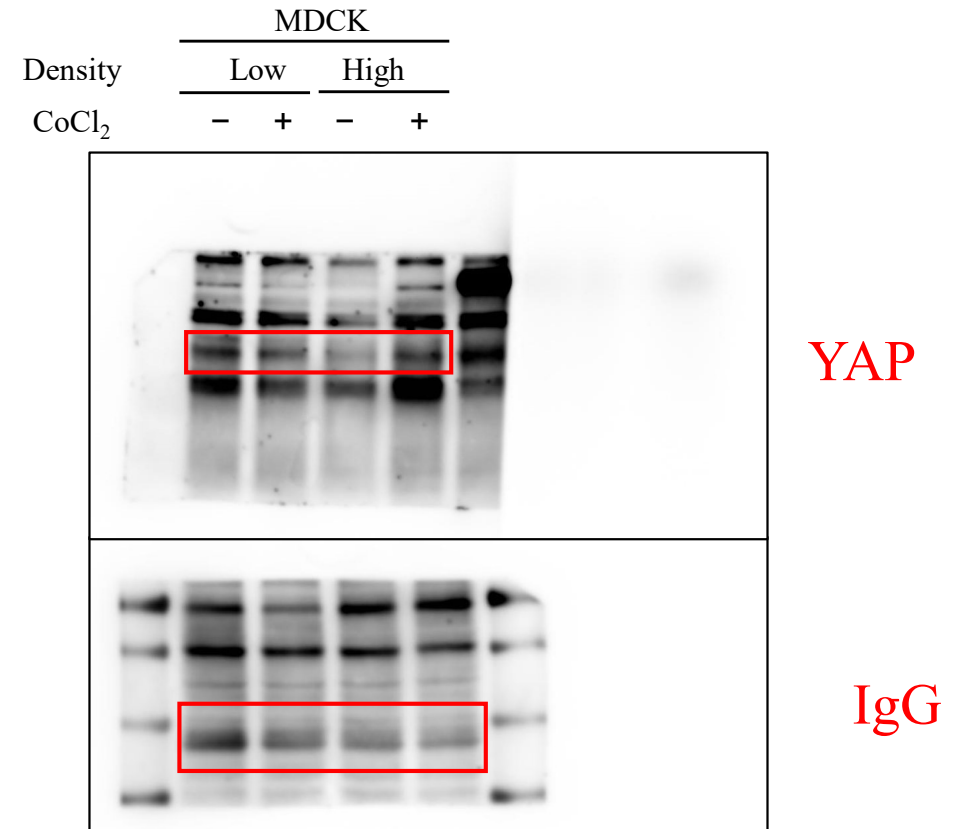

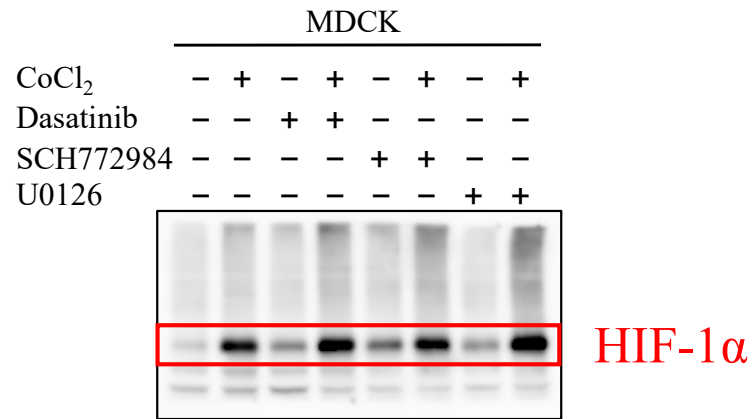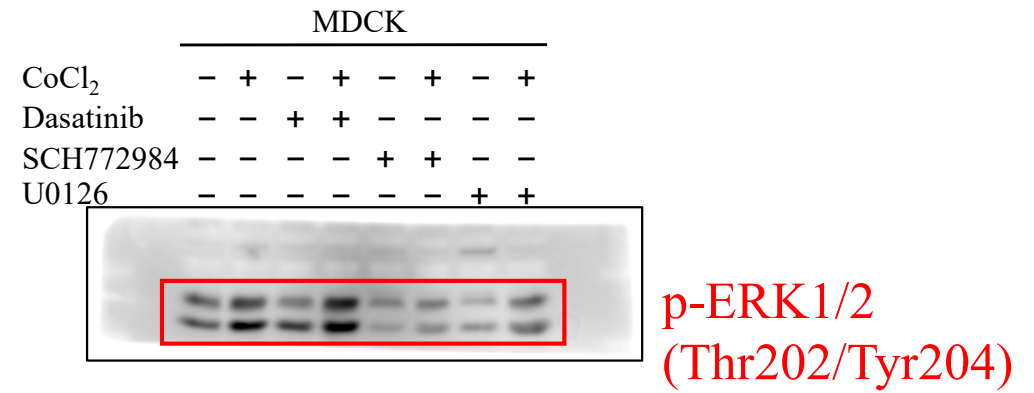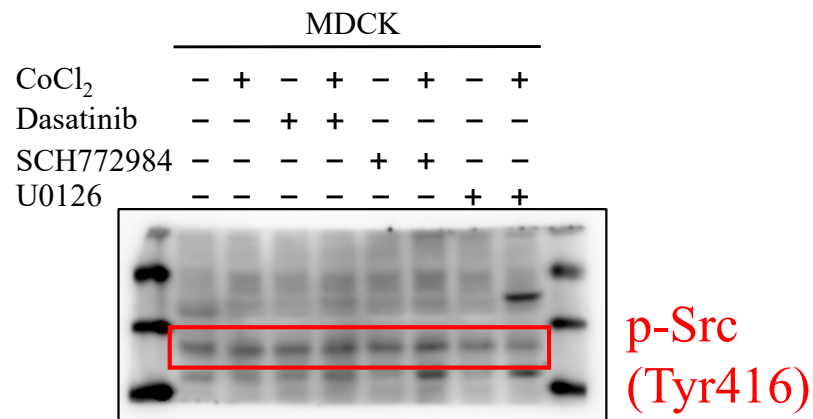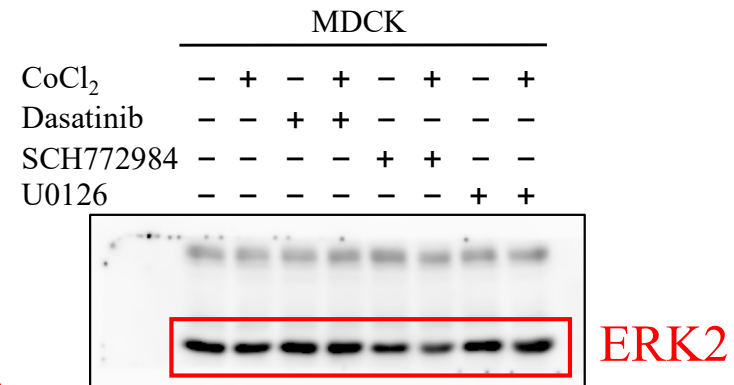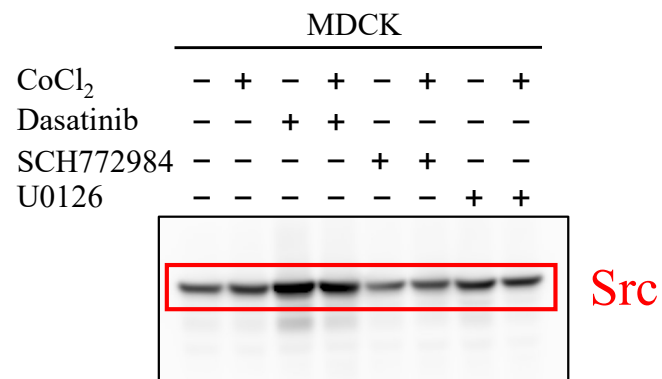

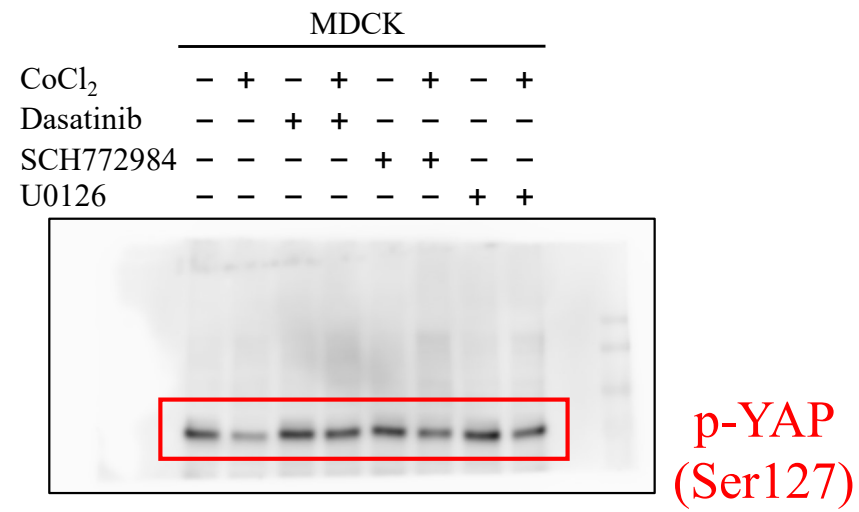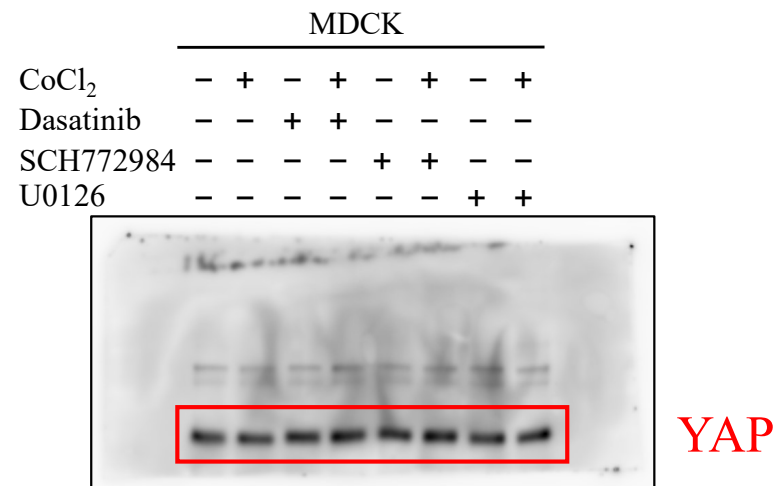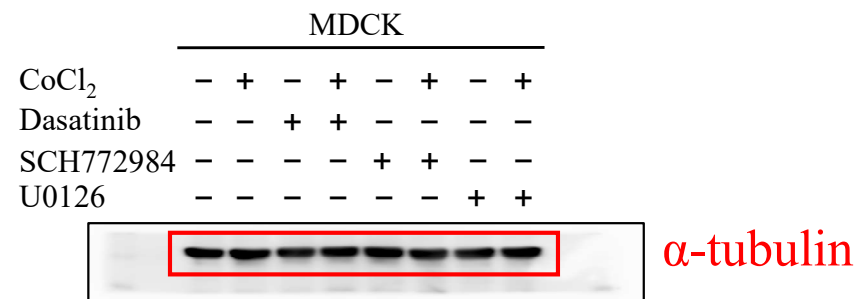

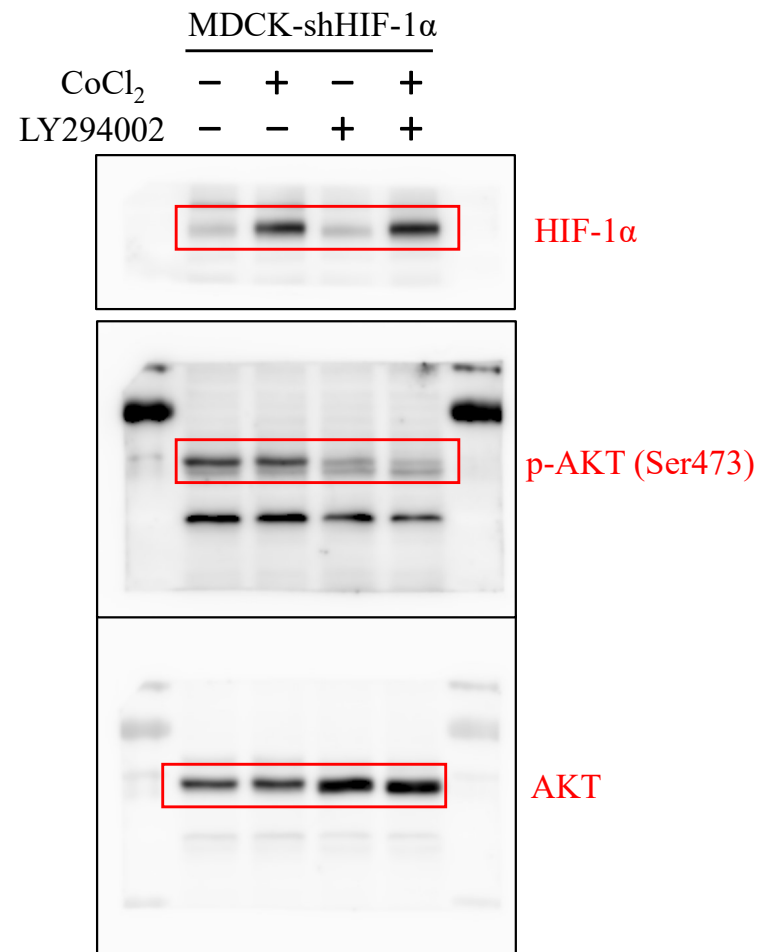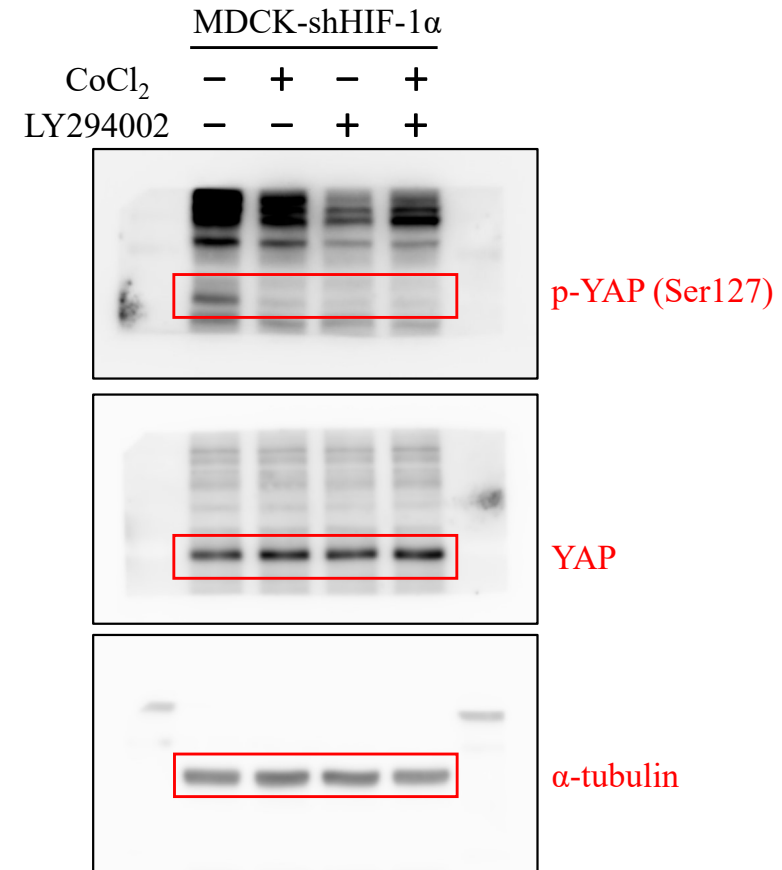

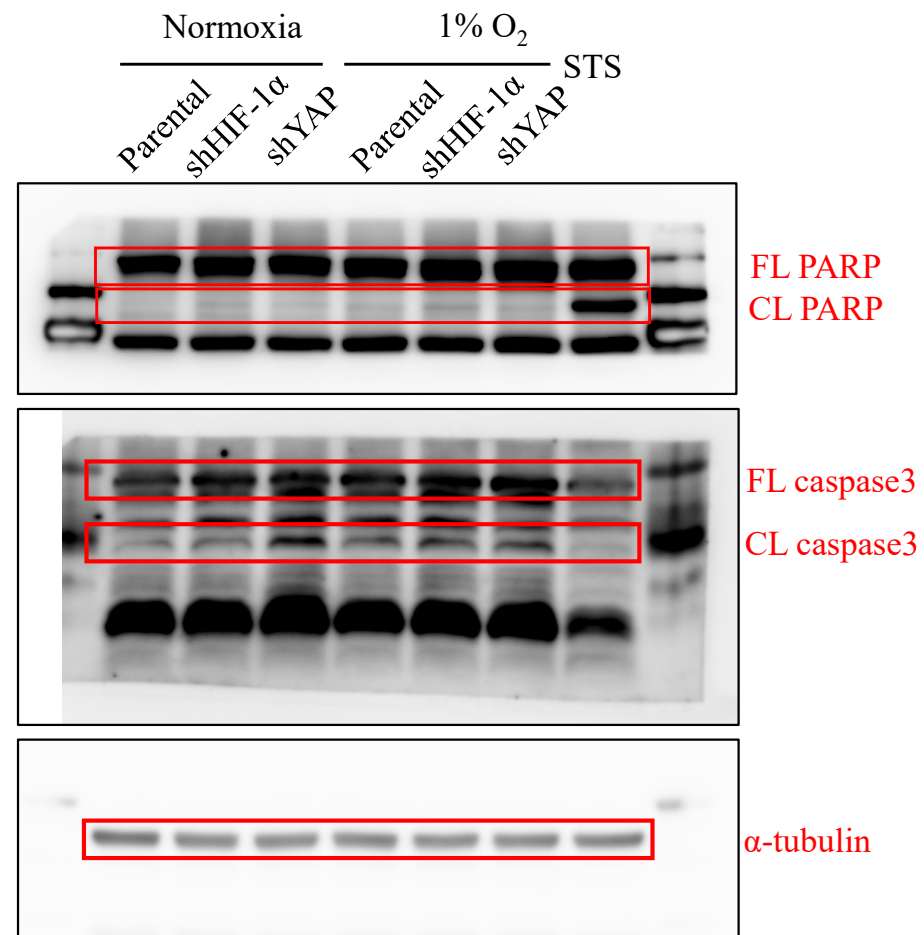

Supplement: Supplementary file 2 — Original data files [file 41420_2023_1687_MOESM2_ESM.pdf]
